# Evaluation of Salt-Tolerant Germplasms and Identification of Salt Tolerance-Related Proteins in Upland Cotton at the Seedling Stage

**DOI:** 10.3390/ijms26051982

**Published:** 2025-02-25

**Authors:** Xiawen Li, Abdul Rehman, Zhenzhen Wang, Hongge Li, Jun Ma, Xiongming Du, Zhen Peng, Shoupu He

**Affiliations:** 1 Zhengzhou Research Base, State Key Laboratory of Cotton Bio-Breeding and Integrated Utilization, School of Agricultural Sciences, Zhengzhou University, Zhengzhou 450001, China; xiawensummer@163.com (X.L.); abdulpbg@gmail.com (A.R.); lihongge@caas.cn (H.L.); 2 State Key Laboratory of Cotton Bio-Breeding and Integrated Utilization, Institute of Cotton Research, Chinese Academy of Agricultural Sciences (ICR, CAAS), Anyang 455000, China; dujeffrey8848@hotmail.com; 3 Research Institute of Economic Crops, Xinjiang Academy of Agricultural Sciences, Urumqi 830091, China; shenhuawzz@163.com (Z.W.); xj.majun@163.com (J.M.); 4 Western Agricultural Research Center, Chinese Academy of Agricultural Sciences, Changji 831100, China

**Keywords:** upland cotton, *Gossypium purpurascens*, TMT-based proteome, salt-tolerant proteins, differential enrichment, biological pathways

## Abstract

Currently, developing cotton cultivation in saline–alkali soils is a vital focus for restructuring the cotton industry in China. The seedling stage, specifically the three-leaf stage, is a crucial period for assessing the salt tolerance of cotton. This research examined 430 natural populations of upland cotton, including 45 semi-wild germlines of *Gossypium purpurascens*. We measured the phenotypic responses of salt stress injury on seedlings as well as potassium (K), calcium (Ca), sodium (Na), and magnesium (Mg) concentrations in the roots, stems, and leaves following a 72 h exposure. The comprehensive salt tolerance index (CSTI) was determined using a membership function, principal component analysis, and cluster analysis based on 48 phenotypic traits related to salt tolerance. The results revealed significant variations in the phenotypic traits of the ion group under salt stress. Salt stress greatly affected the relative contents of Mg, K, and Ca ions in the aboveground parts of cotton, and correlations were observed among the 48 indices. The CSTI was calculated using seven principal component indexes, identifying 30 salt-tolerant, 114 weakly salt-tolerant, 39 salt-sensitive, and 4 highly sensitive materials based on cluster analysis. Among the 45 *G. purpurascens* cotton resources, 28 were weakly salt-tolerant, while 17 were salt-sensitive. Through TMT (Tandem Mass Tag)-based quantitative analysis, we identified 3107 unique peptides among 28,642 detected peptides, resulting in 203,869 secondary mass spectra, with 50,039 spectra successfully matched to peptides. Additionally, we identified several salt tolerance-related pathways (carbon metabolism; glutathione metabolism; the biosynthesis of amino acids, etc.) and proteins classified within the CAZy (Carbohydrate-Active EnZYme) family and expansin proteins. The results of this study concerning salt-tolerant materials provide a crucial theoretical foundation for the identification and evaluation of salt-tolerant breeding parents in cultivated cotton.

## 1. Introduction

Soil salinity is one of the principal abiotic stresses restricting the development of modern agriculture, which will generally lead to a 15~20% reduction in crop yield and quality [1,2]. According to statistics, about 20% of arable land in more than 100 countries around the world is affected by salinization [1,3]. China has approximately 3.69 × 10^7^ hectares of salinized land, representing roughly 5.01% of its total arable land. The ability of a plant to grow and complete its life cycle in saline environments depends on its inherent salt tolerance, which differs notably among various species and developmental stages [4]. Therefore, screening and breeding salt-tolerant crop varieties is a crucial way to deal with the threat of soil salinization. Recent research on crop salt tolerance reveals that salinity disrupts cell membrane permeability through osmotic stress and ion toxicity. This results in oxidative stress and metabolic imbalances, leading to bud rot, root rot, leaf desiccation, and reduced yield [5]. The impact of salt stress on crops varies at different growth stages, with the seedling stage being particularly sensitive. Likewise, the salt tolerance levels of different genotypes differ significantly, both at various growth stages and within the same stage [6]. The salt tolerance at the budding stage was significantly higher than at the seedling stage, making it more practical to study salt tolerance during the seedling stage [7,8].

Upland cotton (*Gossypium hirsutum* L.) is characterized by its extensive cultivation area and superior yield potential, making it the predominant species within the global cotton industry [9,10]. Semi-wild and upland cotton cultivated varieties have a genetic affinity and many excellent traits such as disease and insect resistance, drought resistance, and salinity tolerance [11]. Semi-wild cotton (*Gossypium hirsutum* L. *purpurascens*) is a perennial species native to Hainan. Population genetic analyses indicate that this taxon may have originated along the coastal regions of Mexico [12]. At present, there are few studies on the identification and screening of salt-tolerant materials of wild and semi-wild species of upland cotton. The reduction in arable land and competition between grain and cotton have shifted cotton cultivation practices. Cotton farming is increasingly concentrated in saline–alkali soils, offering significant development opportunities [13]. Saline–alkali stress significantly impacts the absorption and transport of mineral elements in crops. This effect primarily arises from the high concentration of salts in the soil, which leads to ion competition. As a result, the absorption of essential ions in cotton plants is hindered, causing mineral nutrient stress. This stress ultimately disrupts the balance of ion homeostasis in the crops [14]. Research increasingly focuses on how ionic toxicity affects other ions [1]. More than 15% of China’s cotton fields are saline–alkali cotton areas, mainly distributed in the Huang-Huai-Hai Plain and the Southern Xinjiang region [15]. Assessing salt tolerance in cotton germplasms is essential for identifying superior varieties with high tolerance and sensitive types. This approach will enhance genetic diversity and is crucial for optimizing cotton production and ensuring the sustainability of the national cotton industry amid rising soil salinity. At present, the research on the identification of salt tolerance of crop materials is mainly focused on soybean [16], peanut [17], rice [18], wheat [19], mung bean [20], and cabbage [21]. Research on identifying and assessing salt tolerance in upland cotton germplasm resources, particularly for non-edible cash crops, remains limited. Existing studies face several challenges, including an excess of identification indicators without standardized methodologies. Additionally, there is a scarcity of comprehensive and representative germplasm materials, along with an absence of systematic evaluations of salt tolerance across recognized upland cotton varieties.

Recent advancements in proteomic methods have established them as effective techniques for revealing proteomic profiles, thereby enhancing our understanding of plant physiological adaptations to stress [22,23,24]. Additionally, comparative proteomics offers enhanced insights into the molecular mechanisms governing stress responses in plants exhibiting varying degrees of stress sensitivity when subjected to challenging environmental conditions [25,26]. Two-dimensional gel electrophoresis (2-DE) is the main proteomic technique used to identify differentially abundant proteins (DAPs) in alfalfa leaves and roots under abiotic stress, including drought, salinity, and heat treatment [27,28,29,30]. The 2-DE-based proteomic approaches encounter challenges in achieving efficient protein identification and precise quantification [31]. The advancement of proteomics has led to significant improvements in quantitative proteomics, particularly using Tandem Mass Tags (TMTs). This approach enhances both the identification of a more significant number of proteins and the accuracy of their quantification [32,33]. TMTs, or Tandem Mass Tags, are a class of stable isotope-coded chemical labels designed for the multiplexed quantification of peptides. These tags enable the simultaneous assessment of relative protein abundance across two distinct samples, facilitating comparative proteomic analysis through mass spectrometry [34].

In this study, 430 excellent germplasms (including 45 samples of *G. purpurascens*) with a wide range of geographical origins and rich genetic diversity were selected as materials, seedlings were raised by sand culture method, and salt stress treatment was carried out at the seedling stage (three-leaf and one-heart stage) [35]. Phenotypic assessments of potassium (K), calcium (Ca), sodium (Na), and magnesium (Mg) were conducted in roots, stems, and leaves following a 72 h exposure to salt stress. The resulting phenotypic alterations under stress conditions were documented. Subsequently, the distribution and performance variations in the ion groups across different tissues within the population were statistically analyzed using mathematical membership functions, principal component analysis, a comprehensive salt tolerance index, and cluster analysis.

## 2. Results

### 2.1. Classification of Salt Damage Symptoms at Seedling Stage in 430 Upland Cotton Populations

**Figure 1:**
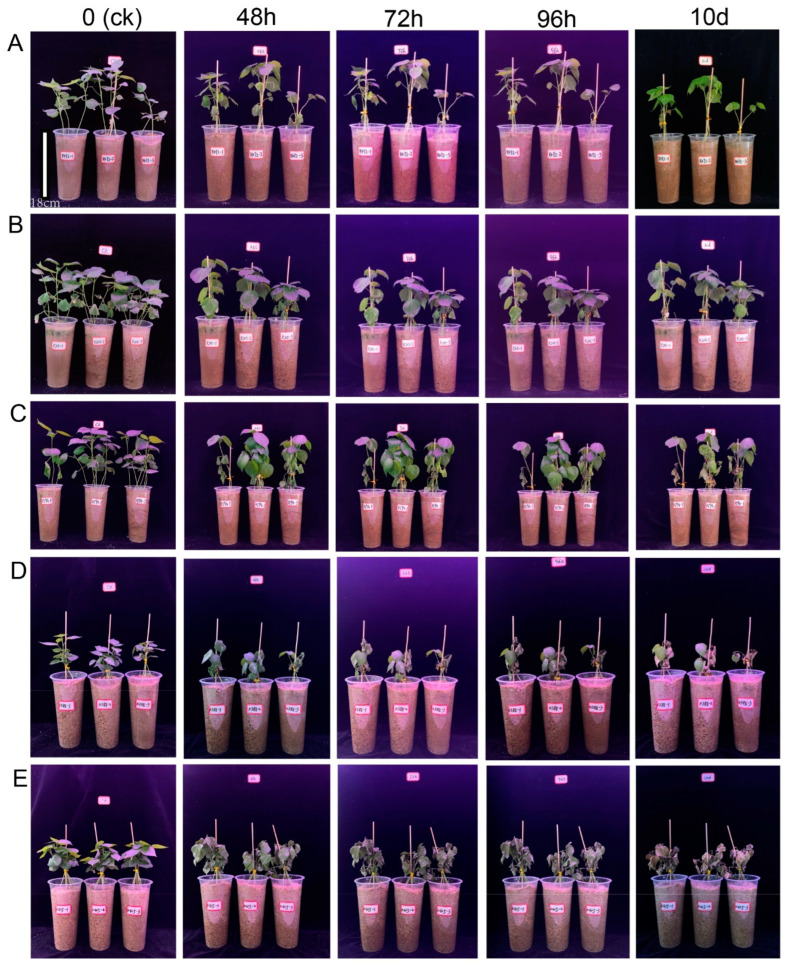
Phenotypic characteristics of different materials at different time points in response to salt stress (0.4% NaCl) under salt stress: (**A**) salt-resistant materials, (**B**) salt-tolerant materials, (**C**) weakly salt-tolerant materials, (**D**) salt-sensitive materials, and (**E**) high-sensitivity salt materials. Bar = 18 cm (cup height).

In the artificial climate room, 430 germplasms from various upland cotton populations, sourced from multiple geographical locations, were grown in sand culture. Once the plants reached the three-leaf and one-heart stage (seedling stage), they were subjected to salt stress treatment [36]. Data were recorded for salt levels before treatment and after salt treatment at different time points (0 h, 48 h, 72 h, 96 h, 10 days) to observe plant phenotypic changes in populations. Based on the salt damage performance of this group at all time points, the degree of salt tolerance was categorized into five grades: A1, A2, A3, A4, and A5 (Figure 1). A1 is a highly salt-tolerant material, and A5 is a highly sensitive material. The phenotypic characteristics of salt damage symptoms and the number of corresponding materials of each grade are shown in Table 1. The results showed that 430 natural populations included 2 salt-resistant materials (A1), 93 salt-tolerant materials (A2), 199 weakly salt-tolerant materials (A3), 92 salt-sensitive materials (A4), and 44 highly sensitive materials (A5). Among the materials examined, those exhibiting weak salt tolerance phenotypes were the most prevalent, comprising nearly 50% of the total. Additionally, the second category of materials, characterized by moderate salt tolerance, represented 21.63% of the overall sample. Phenotypic results showed that the genetic diversity of the 430 upland cotton populations was rich, which reflected the diversity of the materials as well as the clustering results based on genotyping (SNP) (Supplementary Figure S1).

### 2.2. Variation Analysis of Salt Tolerance Coefficient-Related Traits in Ionomics at Seedling Stage of 430 Upland Cotton Populations

The average values of three biological replicates were analyzed and compared using ionome phenotypic data from inductively coupled plasma mass spectrometry. Table S1 shows the descriptive statistics of 48 traits related to Ca, K, Mg, and Na in different parts (leaves, roots, stems, aerial parts, whole plant, and root–shoot ratio) of 430 upland cotton under salt stress. The variation range of 48 traits was 27.71~168.14%. Firstly, 34 indicators with an average value of less than 1 were screened out from all indicators, indicating that the phenotypic values of these 34 traits decreased compared with the control group under salt stress. The coefficient of variation in the 34 traits ranged from 27.71~157.56%, among which the coefficients of variation in RISC-S_R-CavsNa, RISC-Sh_R-Ca/Na, RISC-Sh_R-Mg/Na, ST_S_Ca/Na and RISC-Sh_R-K/Na were more significant, which were 157.56%, 128.28%, 108.01%, 91.82%, and 77.53%, respectively. The results showed that there were large differences between these indicators among different upland cotton varieties. The coefficient of variation in the five indexes of ST_Sh_Mg (27.71%), ST_All_Mg (27.87%), ST_S_Mg (29.29%), ST_L_Mg (30.22%), and ST_Sh_K (31.86%) was small, indicating that these indicators had little influence among different materials. Additionally, this study found that, among the five traits with a large coefficient of variation, K, Ca, and Mg were transferred from the roots to the stem or shoot, while four traits retained Na. This suggests that the roots of this group have a rich and diverse ability to intercept sodium and transport nutrient ions. Nine of the 13 traits with an average value over 1 were linked to Na ions, suggesting a negative correlation with salt tolerance.

The analysis of the 430 upland cotton germplasm lines reveals significant variation in salinity tolerance traits, highlighting substantial genetic diversity within the population. The observed salt tolerance coefficients across different indices indicate diverse responses to salt stress among genotypes. This suggests that reliance on a singular index for evaluating salt tolerance in cotton varieties (lines) is insufficient. Each trait exhibits unique reactions to saline conditions, underscoring the complexity of assessing salt tolerance comprehensively. This analysis of the 430 upland cotton germplasm lines reveals significant variation in salinity tolerance traits, highlighting substantial genetic diversity within the population. The observed salt tolerance coefficients across different indices indicate diverse responses to salt stress among genotypes. This suggests that reliance on a singular index for evaluating salt tolerance in cotton varieties (lines) is insufficient. Each trait exhibits unique reactions to saline conditions, underscoring the complexity of assessing salt tolerance comprehensively [37]. Following exposure to salt stress, all ion-related traits, except sodium (Na), exhibited a decline in their salt tolerance coefficients across all examined tissues. Notably, the K/Na, Ca/Na, and Mg/Na indices in the five tissue types (root, stem, shoot, leaf, and overall) demonstrated heightened sensitivity to salinity conditions. In contrast, the salt tolerance coefficients for Ca and Mg ions across various tissues showed relatively minimal impact from salt stress.

### 2.3. Correlation Analysis of 430 Traits Under Salt Stress at Seedling Stage of Upland Cotton

**Figure 2:**
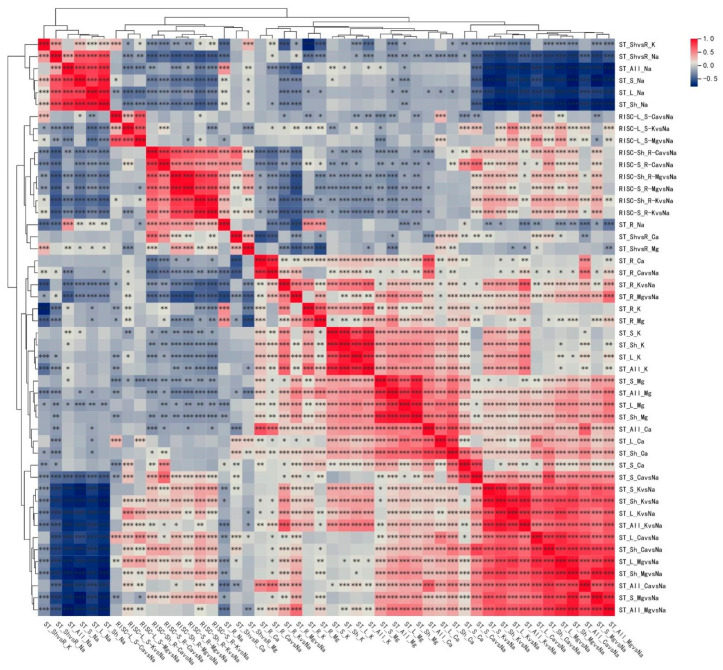
Heat map of the correlation between salt tolerance coefficients of 48 ionome-related traits. Note: *, ** and *** represented *p*-value < 0.05, <0.01, <0.001, respectively.

The relative values (salt tolerance coefficients) of 48 ionome-related traits were analyzed by TBtools-II software (v2.056), as shown in Figure 2. The findings indicated a robust correlation between the salt tolerance coefficients of various plant tissues assessed under identical conditions. From an ionic perspective, the salt tolerance coefficient for sodium ions Na exhibited a significant relationship with K, Ca, and Mg ions. The ratios of K(Ca,Mg)/Na displayed low or negative correlations with the salt tolerance coefficient of selective transport and a significant negative correlation with the salt tolerance coefficient from the K(Ca,Mg)/Na ratio. In examining the root-to-shoot ratios of various ions, the salt tolerance coefficients for K, Ca, and Mg reveal that the K ion’s root/shoot ratio strongly correlated with the Na ion salt tolerance coefficient.

Additionally, there was a significant positive correlation between the salt tolerance coefficient of the Mg root/shoot ratio and the salt tolerance of Na in the leaf tissues (including stems and other aerial parts). Furthermore, there were notable correlations with the salt tolerance coefficients for the selective transport of Ca/Na from stem to leaf and for K/Na from root to stem. The salt tolerance coefficient of K/Na in leaves, roots, stems, aerial parts, and whole plants strongly correlates with the salt tolerance coefficients of Ca/Na and Mg/Na. Additionally, it was positively correlated with the K ion salt tolerance coefficient in those same plant parts. In contrast, it negatively correlated with sodium ions’ salt tolerance coefficient in leaves, roots, stems, aerial parts, and whole plants. The correlation analysis results in Table S2 indicate that this study’s indicators were interrelated and consistent, revealing significant redundancy in the information provided. Consequently, it is essential to streamline the index system by employing principal component analysis to consolidate these metrics effectively.

### 2.4. Analysis of Significant Differences in Salt Tolerance Coefficient Between G. purpurascens Cotton and Cultivated Upland Cotton

**Figure 3:**
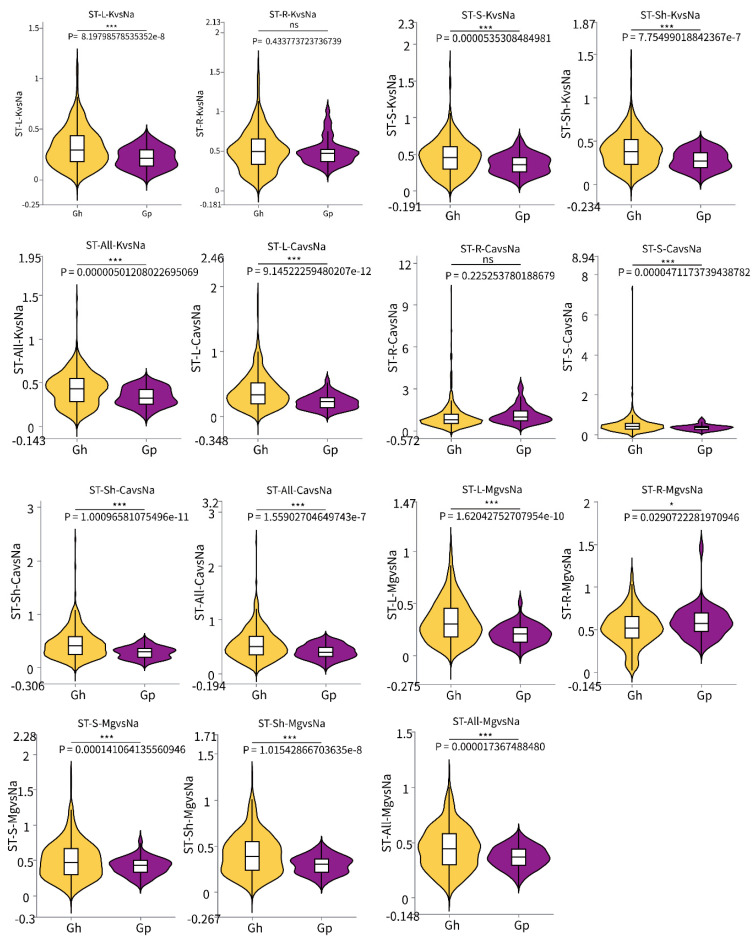
Significant differences in the salt tolerance coefficient indexes of K/Na, Ca/Na and Mg/Na ion ratios between the two varieties in different parts. Note: ns, * and *** represented *p*-value > 0.05, <0.05, <0.001, respectively.

The salt tolerance coefficients of 385 cultivated upland cotton cultivars and 45 semi-wild cotton cultivars (*G. purpurascens*) were analyzed across different tissues (Figure 3). Additionally, the Ca, K, Mg, and Na ion concentrations in these tissues were examined using GraphPad Prism 9.0.0 software (Supplementary Figure S2). The selective transport coefficient, expressed as K(Ca,Mg)/Na, was also evaluated from root-to-shoot, stem-to-leaf, and root-to-stem (Supplementary Figure S3). The boxplot results revealed that the salt tolerance coefficients, specifically the K/Na and Ca/Na ratios, were significantly higher in the leaves, stems, shoots, and whole plants compared to *G. purpurascens*. However, no differences were observed in the roots. The Mg/Na ratio was significantly higher in the leaves, stems, shoots, and whole plant of upland cotton than *G. purpurascens*. In contrast, the content in the roots of cultivated upland cotton was significantly lower than *G. purpurascens*. It was suggested that *G. purpurascens* was more sensitive to salt stress than upland cotton (Figure 3). There was no significant difference in the salt tolerance coefficients of Na and K ion content in the roots of *G. purpurascens* compared to cultivated upland cotton. The contents of K, Mg, and Na ions in the leaf, stem, aerial part and whole plant of *G. purpurascens* were significantly higher than in upland cotton (Supplementary Figure S2). In comparing the salt tolerance coefficients of ion-selective transport, the RISC values from root-to-shoot, root-to-stem, and stem-to-leaf of cultivated upland cotton were significantly higher than those of *G. purpurascens*. This indicates that, after experiencing salt stress, the transport capacity of seedling roots to different parts of the plant was stronger in cultivated upland cotton than in the *G. purpurascens* population (Supplementary Figure S4). The findings indicate that the salt tolerance observed in upland cotton genotypes is generally superior to that of *G. purpurascens*.

### 2.5. Principal Component Analysis of 430 Offspring Cotton Traits Under Salt Stress at Seedling Stage

To reduce the redundancy of the data, the original index was replaced with fewer principal components, and the principal component analysis was carried out on each index. The eigenvalues of each principal component factor, the load matrix to the original index, and the contribution rate to the phenotype are shown in the table. According to the PCA, the cumulative contribution rate exceeds 80%. Therefore, the first seven principal component factors were selected, with the following contribution rates: 29.38%, 17.09%, 9.38%, 7.50%, 6.39%, 5.55%, and 5.13%. The cumulative contribution rate of these factors reached 80.43%, effectively representing most of the information from the 48 indicators. Table S3 showed that the principal component factor F1 was primarily associated with the magnesium-to-sodium (Mg/Na) salt tolerance coefficients in the shoots, whole plants, and leaves of the upland cotton population. The principal component factor F2 was mainly related to the selective transport coefficients of the ion ratios Mg/Na, K/Na, and Ca/Na as they move from the roots to the stem and from the roots to the shoots. Principal component factor F3 primarily influences the salt tolerance coefficient of K in the shoots, whole plants, stems, and leaves. Principal component factor F4 was mainly associated with the salt tolerance coefficients of Mg in the shoots and stems. The principal component factor F5 was primarily associated with the salt tolerance coefficients of K and Mg ions in the roots and the salt tolerance coefficient of the Na ion root-to-shoot ratio and other related traits. Principal component factor F6 was closely linked to the salt tolerance coefficients of Ca ions in the roots and the whole plant and the Ca/Na ratio in the roots and the entire plant. Principal component factor F7 was linked to the salt tolerance coefficients of Ca in the plant’s stems and aerial parts, the Ca/Na ratio, and the salt tolerance from root-to-stem and aerial parts. This factor also showed a close relationship with the selective transport coefficient of K and the salt tolerance coefficient of Na from the stem to the leaf. In summary, 48 salt tolerance-related indices from 430 upland cotton populations were consolidated into 7 independent comprehensive indices through principal component analysis for the subsequent evaluation and analysis of salt tolerance.

### 2.6. Evaluation of Salt Tolerance at Seedling Stage of 430 Upland Cotton Populations

The membership function values for the seven composite indexes were calculated using the principal component values of various cotton genotypes, as indicated by Formula (9). Subsequently, the weights of these seven composite indexes were determined based on the contribution rates of the principal component factors, according to Formula (11). The contribution rates for the seven principal component factors were as follows: 0.365, 0.212, 0.117, 0.093, 0.079, 0.069, and 0.064, respectively. After determining the comprehensive index’s membership function values and weights, the CSTI value for the overall evaluation of salt tolerance was calculated using Formula (12). Afterwards, the salt tolerance of 430 upland cotton germplasms was ranked based on the CSTI values (Table S4). The results showed that, among the 430 germplasms of upland cotton populations, the top 10 varieties with the highest CSTI were K126 (Xiaoxian 133 long-staple), K069 (Ejing 92), K142 (Jinmian 2), K055 (Xinxian-Lu), K143 (Chirpan 996), K202 (E901), K120 (Zhong 521), K194 (Ganzao 032), K260 (Jinmian 34), and K244 (Bao 2367). The bottom 10 with a low salt tolerance index were K379 (Yinshan No. 6), K328 (N73DeltapineNGF), K323 (108F), K329 (Zhong 21371), K327 (FH682), K324 (Sizimian 453), K325 (gL2gl3), K290 (Bukhara No. 6), K010 (Jinmian 27 (Yuan 2918)), and K011 (AC-241).

**Figure 4:**
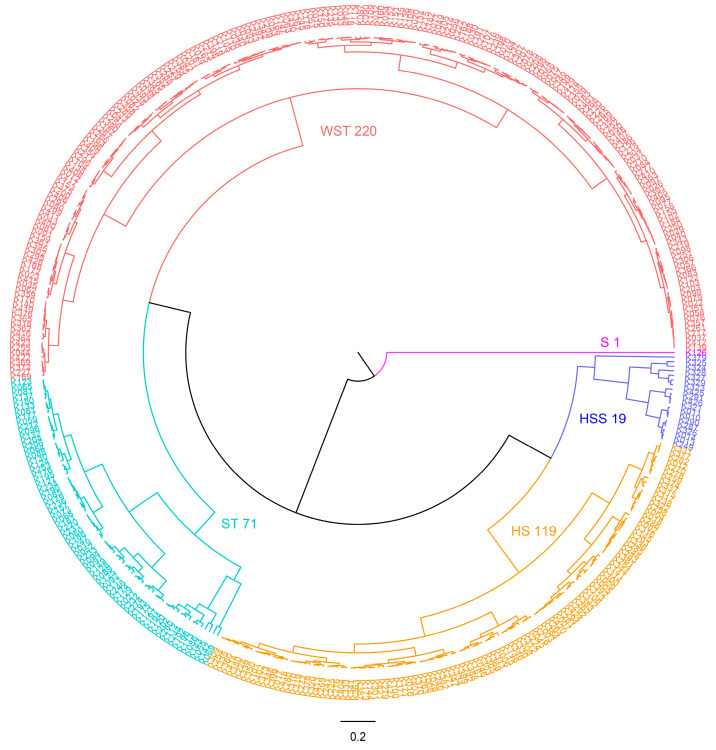
Cluster map of 430 upland cotton germplasms based on CSTI values.

Based on the CSTI values of various upland cotton varieties, we used the shortest distance method in systematic cluster analysis to categorize these varieties (Figure 4). As a result, 430 upland cotton varieties were classified into five distinct grades (Table 2): L1. The first grade consists of salt-resistant varieties, with only one identified variety exhibiting a high CSTI value of 5.633; L2. The second grade includes 71 varieties categorized as salt-tolerant, with CSTI values ranging from 3.652 to 5.108; L3. Third grade varieties were classified as having weak salt tolerance, comprising 220 varieties with CSTI values from 2.503 to 3.629; L4. The fourth grade grouping contains 119 salt-sensitive varieties, with CSTI values between 1.563 and 2.478; L5. Finally, the fifth grade consisted of 19 highly sensitive varieties, exhibiting CSTI values that fall within the range of 0.411 to 1.495. This classification framework aids in identifying and understanding the salt tolerance capabilities of different varieties, which is crucial for agricultural and ecological applications in saline environments. Out of the 45 germplasms of *G. purpurascens* examined, all exhibited weak salt tolerance or were classified as salt-sensitive and highly sensitive. More than half of the germplasms, approximately 62.22%, demonstrated weak salt tolerance, while 37.78% were identified as salt-sensitive or highly sensitive materials. Among the 430 germplasms, 67.67% of the CSTI materials were classified as salt-tolerant or weakly salt-tolerant, while approximately 32.09% were identified as salt-sensitive or highly sensitive materials.

Additionally, a correlation was found between CSTI values and the salt tolerance coefficients of 48 indexes (Table S2). There was a significant correlation between the relative Ca content in shoots and the relative Ca content in the entire plant, as well as between the K content in shoots and the whole plant. The relative Na content in leaves was also significantly correlated, with all correlations exceeding 0.5. Other correlations were significant but lower; these included the relative Ca content in stems, K content in leaves and stems, Mg content in leaves, stems, shoots, and the whole plant, and Na content in stems, shoots, and the whole plant. These correlations all exceeded 0.4 but were less than 0.5. Among the 48 evaluated indicators, the 15 highlighted indicators significantly influence the salt tolerance assessment during the seedling stage of upland cotton. Therefore, under the same conditions, these 15 indicators can be regarded as essential indicators for the rapid evaluation of salt tolerance of upland cotton germplasms.

### 2.7. Salt Tolerance Identification Results of 430 Upland Cotton Germplasms

The analysis of salt damage phenotypes at the seedling stage, in combination with the ionomic evaluation and grading of 430 germplasm accessions, enabled a cross-comparative assessment of salt-tolerant varieties (Table 3). The 430 germplasm materials were categorized into five grades based on their salt response: salt resistance, salt tolerance, weak salt tolerance, salt sensitivity, and high sensitivity. Within this classification, A1 to A5 represent the phenotypic results of salt damage, while L1 to L5 indicate the clustering results based on ion group analysis. The salt tolerance of 430 upland cotton germplasms was evaluated, and the corresponding varieties are listed in Table S4. We discovered 30 salt-tolerant varieties using two methods. These include Ejing 92, Jinmian 2, Chirpan 996, and E901. These varieties represent 42.3% of the materials classified by ionome and 32.3% of the materials classified by salt damage phenotype. A total of 114 weakly salt-tolerant varieties, including Jinmian 49, Nongdamian 7, and Yuzao 275, account for 51.8% of the ionomic classification and 57.3% of the salt damage phenotypic classification. A total of 39 salt-sensitive varieties, including Taiyuan 02-47, Jimian 11, Zhanjiang Jinzhou Jialumian 2, and Kuaiche Express, account for 32.8% and 42.4% of the ionomic classification and salt damage phenotypic classification, respectively. There were four corresponding high-sensitivity salt varieties, including Z37 less, Jinmian 27 (Yuan 2918), Bukhara 6, and Yinshan 6, which accounted for 21.1% and 9.1% of the ionomic phenotypic evaluation and salt damage phenotypic classification, respectively. Even if the varieties did not correspond to both methods simultaneously, there was still some reference value for assessing salt tolerance through ionomic cluster evaluation or phenotypic classification of salt damage. For example, of the 19 materials classified by ionomic cluster evaluation, 16 were identified as salt-sensitive or highly sensitive varieties, including highly sensitive varieties and special island landraces, such as Jialu-7, which exhibited salt damage.

A total of 187 exhibited salt tolerance, representing 43.5% of the total, out of 430 analyzed upland cotton materials. This assessment was based on two methodologies: ionomic phenotypic evaluation and salt damage phenotype classification. The results concerning salt tolerance from these materials are highly reliable, providing valuable insights for selecting and breeding superior varieties with enhanced adaptability.

### 2.8. Quantitative Proteomic Responses to Salt Stress

LC-MS/MS analyzed the samples, and 3107 proteins were identified (Supplementary Table S5). A total of 28,642 peptides were identified, with 203,869 total secondary mass spectra, of which 50,039 were matched to peptides (24.54% spectral utilization rate).

**Figure 5:**
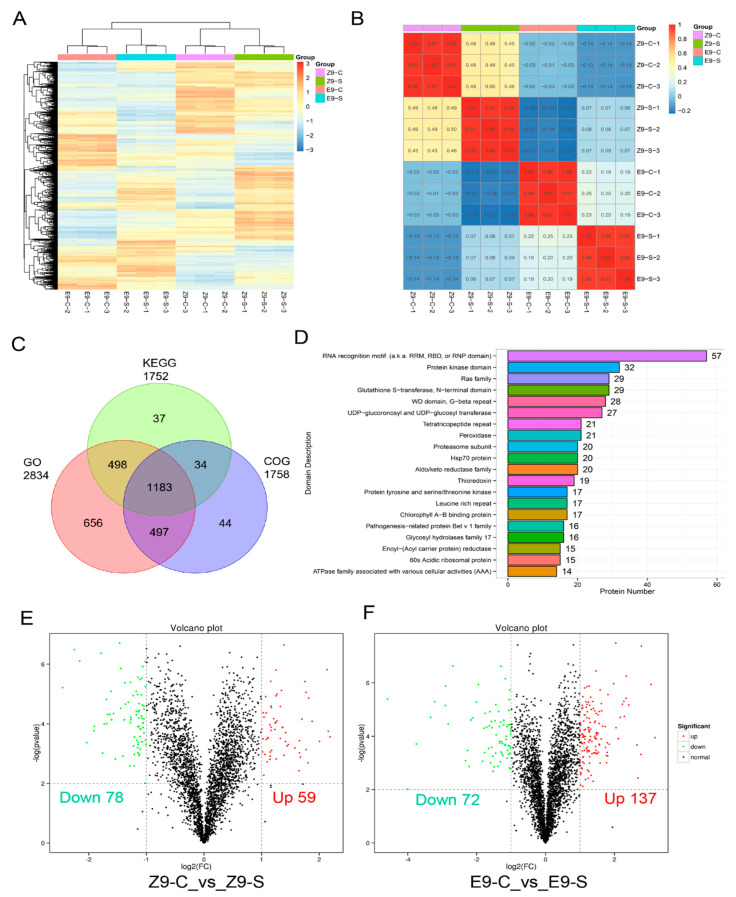
Protein identification from TMT proteomics: (**A**) heatmap of all protein expression levels, (**B**) correlation of 12 samples, (**C**) database annotation of all proteins, (**D**) Pfam database comparison, (**E**) volcano plot of differentially expressed proteins in Z9, and (**F**) volcano plot of differentially expressed proteins in E9.

The relative quantification of reliable proteins yielded expression data for all 3107 proteins (Supplementary Table S6). A heatmap was generated to visualize expression changes in the same protein across samples before and after stress (Figure 5A). Results showed good reproducibility among the three biological replicates within each treatment group but significant differences between the control (C) and stress (S) groups. Correlation analysis of 12 samples showed over 95% correlation among replicates in the same treatment group but lower correlation between C and S groups, particularly for salt-tolerant samples (Figure 5B). Functional annotation of the 3107 identified proteins was conducted using Gene Ontology (GO), the Kyoto Encyclopedia of Genes and Genomes (KEGG), and Cluster of Orthologous Groups (COG) databases to elucidate their functional characteristics. A total of 2834, 1752, and 1758 functional proteins were annotated in GO, KEGG, and COG databases, respectively, with 1183 proteins identified in all three categories (Figure 5C). Pfam was employed to annotate 3107 protein sequences (refer to Figure 5D), facilitating insights into their structural and functional characteristics. The RNA binding (RRM/RBD/RNP motif) family had the most members (57) involved in RNA metabolism (splicing, transport, translation, and stability). Additional families associated with plant stress resistance encompass the Protein Kinase domain (32), Glutathione S-transferase N-terminal domain (29), Ras superfamily (29), WD repeat domain (G-beta repeat) (28), and UDP-glucuronic acid and UDP-glucose transferases (27).

We selected two standard sample pairs for comparative evaluation in the final analysis. The fold change was determined by calculating the ratio of the mean quantitative values of all replicates for each protein within the compared pairs. The statistical significance was determined using a *t*-test (*p*-value < 0.05). The criteria for differential screening included a fold change threshold of ≥2 or ≤0.5. Results regarding the differential protein analysis are presented in Figure 5E,F. Salt stress resulted in 137 differentially expressed proteins in the salt-tolerant control material (Z9) (59 up-regulated, 78 down-regulated), and 209 in the salt-tolerant material (E9) (137 up-regulated, 72 down-regulated). E9 showed more up-regulated than down-regulated proteins, while Z9 showed the opposite. This suggests that the more resistant material (E9) actively responds to salt stress by increasing the activity of resistance-related proteins to enhance immunity while also reducing “activity” (down-regulating proteins) to withstand the adverse environment.

### 2.9. Enrichment of DAPs by KEGG Pathway and Identification of Salt Tolerance-Related Proteins Under Salt Stress

**Figure 6:**
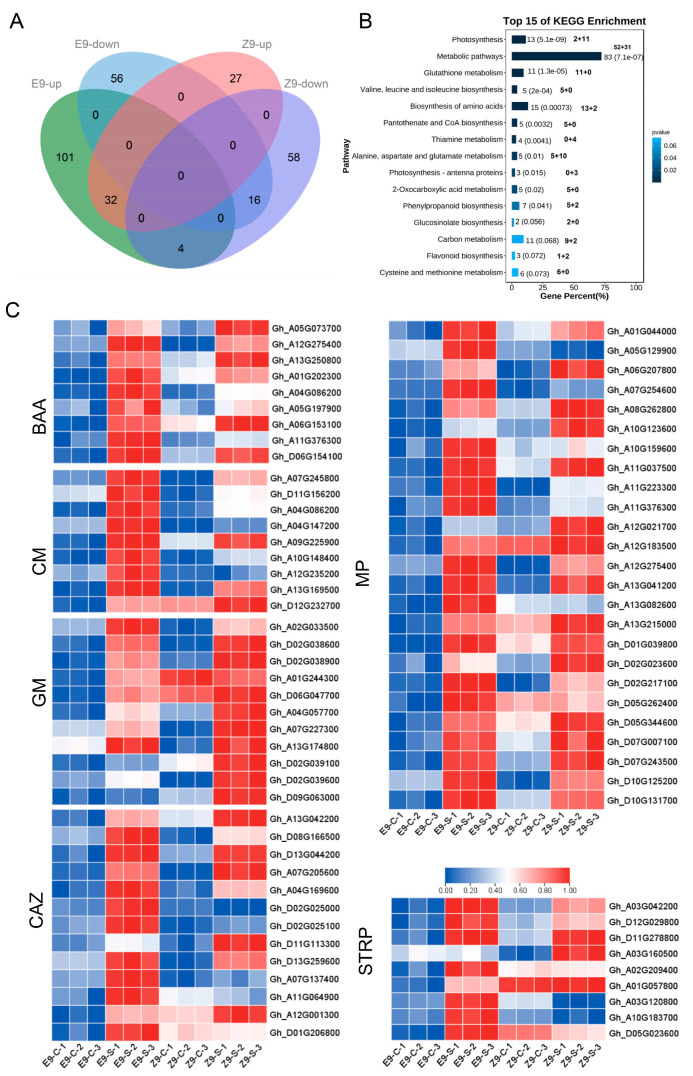
Analysis of differentially expressed protein data: (**A**) Venn diagram showing the intersection of up- and down-regulated differentially expressed proteins in the two materials before and after stress, (**B**) the top 15 KEGG pathways of up- and down-regulated proteins, and (**C**) the heatmap of expression levels of up-regulated proteins in enriched KEGG pathways.

Up- and down-regulated proteins from both materials were compared. E9 had more uniquely up-regulated proteins (101) than Z9 (27) (Figure 6A). Since both materials are salt-tolerant, their differentially expressed proteins are considered salt tolerance-related. A total of 164 proteins were up-regulated (101 + 32 + 4 + 27), and 130 were down-regulated (56 + 58 + 16). KEGG pathway enrichment analysis was performed on these salt tolerance-related proteins (Supplementary Table S7). The top 15 KEGG pathways are shown (Figure 6B), with each bar representing a pathway and showing the number of up (left) and down-regulated (right) proteins.

Figure 6B shows that the main enriched pathways (*p* < 0.05) were metabolic pathways (MPs), the biosynthesis of amino acids (BBA), glutathione metabolism (GM), and carbon metabolism (CM) (ranked by the number of up-regulated proteins). Heatmaps of expression levels of all up-regulated proteins in these KEGG pathways were generated. Some salt tolerance-related proteins and other potentially related proteins were also identified as members of the CAZy (Carbohydrate-Active enZYme Database) family. Heatmaps showing their expression in the 12 samples of both materials were generated (Figure 6C; Supplementary Table S8). Most of these proteins showed a sharp increase in expression after salt stress in E9, with a smaller rise in Z9, reflecting the more substantial salt tolerance of E9. The coordinated up-and down-regulation of these 304 identified salt tolerance-related proteins contributes to adaptation to salt stress.

## 3. Discussion

Soil salinization is a global problem that restricts crop production [38]. Therefore, exploring crop tolerance to salt stress can identify valuable variants and genes associated with salt tolerance, which is essential for addressing the conflict between salinized land and arable land [39,40]. Research indicates that the optimal phase for assessing salt tolerance in cotton germplasms occurs during the seedling and flowering and boll development stages. Salinity stress is particularly critical during the early seedling period, specifically at the three-leaf and one-heart stages. Seedlings are germinating and differentiating, with small bolls and tender tissue, making this stage highly susceptible to salinity effects [41,42].

This study investigated 430 natural populations of upland cotton, including 45 semi-wild germlines of *G. perpurascens*. We measured the phenotypic responses of K, Ca, Na, and Mg concentrations in the roots and stems after exposure to a 0.4% salt stress. In the past decade, there has been no study on the relationship between key ion absorption, transport, and salt tolerance after salt stress in large groups of cotton germplasm resources. Numerous studies have indicated that a salt concentration of 0.4% represents the threshold for phenotypic differentiation in cotton seedlings during the seedling stage [35,43]. This experiment focused on Ca, K, Mg, and Na ions in cotton leaves, roots, stems, and shoots. It analyzed the relative content of these ions in different parts of the plant using the membership function method and principal component analysis. A total of 48 indices were collected, including the salt tolerance coefficients for four ions in various parts of the plant. Additionally, we analyzed the salt tolerance ratios of K/Na, Ca/Na, and Mg/Na in different regions. We also measured the selective transport coefficients from the root to the stem, from the stem to the leaf, and from the root to the shoot, consistent with the previous findings [44,45,46]. Research revealed that salt stress affects nutrient uptake in crops, causing deficiencies. Salt-tolerant cotton effectively absorbs K, Ca, and Mg, blocking sodium transport to leaves, which helps it thrive in saline conditions. Maintaining a K/Na balance is vital for its salt tolerance [45,46]. In addition, these inorganic ions play a critical role in enhancing the salt tolerance of plants. Specifically, ions such as Na^+^, K^+^, Cl^−^, Ca^2+^, and Mg^2+^ contribute significantly to the physiological processes that enable plants to withstand saline environments. Their presence helps maintain osmotic balance, supports cellular functions, and aids in various metabolic activities essential for plant survival under salt stress [8,47,48]. Moreover, the impact of salt stress on plants encompasses ionic stress, deficiencies in mineral elements, osmotic stress, and other mechanisms [43,44,45]. Another research study also indicated that reconstructing the ion homeostasis mechanism under saline–alkali stress is crucial to enhancing plant salinity tolerance [43]. The phenotypic changes due to salt damage in all varieties were graded before and after salt stress. The results of ionome phenotypic clustering were analyzed to identify reliable salt-tolerant and salt-sensitive cotton germplasms. In alignment with our results, researchers systematically classified 346 mung bean genotypes based on economic clustering and associated phenotypic variations [20].

A total of 48 traits, including Ca, K, Mg, and sodium Na salt tolerance coefficients, were analyzed across 430 distinct germplasm materials. This suggests a rich genetic diversity among the examined germplasms, highlighting their potential for further exploration and utilization in breeding programs following the prior research [49,50]. The analysis of the CSTI indicated that the relative contents of Ca, K, Mg, and Na in the leaf tissues were the most closely correlated with salt tolerance. This evaluation focused on three tissues from the aerial parts of the plant: the stem, the aerial parts, and the leaves. Likewise, research on watermelon at the seedling stage also verified that Na, Ca, and Mg contents were related to salt tolerance [51]. We assessed CSTI’s efficacy in evaluating salt tolerance in 430 upland cotton seedlings using 48 specific indices during the seedling phase. These materials were categorized into five grades based on their salt tolerance: salt-resistant, salt-tolerant, weakly salt-tolerant, salt-sensitive, and highly sensitive. This classification provides an objective assessment of the salt tolerance levels within the germplasm population. A comparative analysis of salt damage in different plant varieties showed that more than fifty percent of the weakly salt-tolerant varieties in the third grade had significant overlap between pre- and post-salt stress treatments. This suggests a consistent response pattern to salt stress among the evaluated materials. Likewise, the second grade of salt tolerance and the fourth grade of salt sensitivity represented over a third of their respective categories. Notably, the high sensitivity and high salt tolerance grades exhibited minimal to no overlap between the two assessment methods. Despite the limited number of materials in the first and fifth grades, this does not eliminate the possibility of more significant variation among the highly salt-tolerant or highly sensitive materials themselves. In alignment with our results, the researchers classified wheat and rice into five distinct categories based on their tolerance to salt, ranging from highly tolerant to highly susceptible [52,53]. In the CSTI cluster evaluation, 62.22% of the 45 samples showed weak salt tolerance, while the rest were salt-sensitive or highly sensitive, primarily in phenotypic classifications A3, A4, and A5. Among 430 upland cotton samples, 67.67% were either salt-tolerant or weakly tolerant, with the remainder identified as sensitive. Both grading methods revealed varying degrees of salt tolerance, from weak to high sensitivity, consistent with ionomic phenotypic evaluations. Previous researchers also used CSTI values to select salt-tolerant and salt-sensitive genotypes in cotton, wheat, and chickpea [54,55,56]. Preliminary assessments indicate that the salt tolerance exhibited by the 45 *G. purpurascens* germplasms during the seedling stage is inferior to that of the 385 cultivated upland cotton populations. The primary classification of these germplasms ranges from weak salt tolerance to significant salt sensitivity, with many displaying pronounced sensitivity to saline conditions. The current findings contradict our earlier prediction regarding the high salt tolerance and early identification of *G. purpurascens*, which we attributed to its native habitat [40]. This inconsistency may stem from the choice of control materials and the limitations of the existing indoor neutral salt stress testing protocol [40].

Using TMT-based quantitative analysis, 3107 and 28,642 peptides were identified, with 203,869 secondary mass spectra, of which 50,039 were matched to peptides. Among them, 130 proteins were modulated by salt stress in both cotton varieties (Figure 6A). An analysis of key DAPs and their primary involvement pathways was conducted utilizing the KEGG database. The results revealed significant proteomic-level differences in the salinity stress responses between the two cotton varieties. A comprehensive analysis identified 73 distinct metabolic pathways, with the four most significantly enriched categories among the differentially expressed proteins (DEPs) being metabolic pathways (MP), the biosynthesis of amino acids (BBA), glutathione metabolism (GM), and carbon metabolism (CM) (Figure 6B). Prior research has forecasted numerous peptide transporters and receptors within plant systems [57,58]. The scarcity of bioactive peptides in plants could be attributed to the limited utilization of peptidomic methodologies within plant science research [59]. At present, there is a lack of a comprehensive and reliable database for the annotation of plant peptides. The proteolytic cleavage of precursor proteins represents a crucial mechanism for generating these peptides [60,61]. The examination of precursor proteins using GO and the KEGG can provide insights into the origin and potential biological roles of associated peptides. KEGG analysis showed that DEP precursor proteins in leaf tissues were mainly linked to metabolic pathways, the biosynthesis of amino acids, glutathione metabolism, and carbon metabolism. All pathways are crucial for plant growth and material accumulation, and plant stress responses are closely coordinated with growth-related processes [62]. Following exposure to saline stress, plants exhibit a series of physiological and biochemical adjustments that facilitate acclimatization to harsh conditions. This involves the modulation of metabolic pathways and changes in gene expression, enabling the plants to maintain homeostasis and optimize physiological function under osmotic stress. The functions of DAP precursor proteins were linked to plant resistance to salt stress, indicating their role in cotton seedling resilience. The findings demonstrate a significant enrichment of proteins related to the starch and sucrose metabolic pathway in leaf tissues exposed to salt stress, highlighting the critical role of these metabolic pathways in plant responses to saline conditions. Carbohydrates function as energy reserves in the form of polysaccharides like starch and disaccharides such as sucrose. These compounds play a critical role in supporting metabolic processes and are vital for the physiological survival of plants [63,64].

A total of 2834, 1752, and 1758 functional proteins were annotated in the GO, KEGG, and COG databases, respectively, with 1183 proteins common to all three (Figure 5C). Pfam annotated 3107 protein sequences (Figure 5D), revealing insights into their structural and functional traits. The RNA binding (RRM/RBD/RNP motif) family contained the most members, linked to RNA metabolism. Other families related to plant stress resistance included the Protein Kinase domain, Glutathione S-transferase N-terminal domain, Ras superfamily, WD repeat domain, and UDP-glucuronic acid/glucose transferases. Remarkably, each gene family that we identified has been documented in previous research as playing a crucial role in enhancing salinity tolerance across a range of crop species. This connection reinforces the validity of our findings, suggesting a well-established link between these gene families and the ability of plants to withstand saline environments [65,66,67]. Moreover, salt tolerance-related proteins were also identified as CAZy family members and Expansin proteins. Most of the genes of this family exhibited a sharp increase in expression patterns, revealing that these genes are involved in the salt tolerance of cotton. A significant number of members within the CAZy family have been previously documented to play a role in salt tolerance mechanisms across various crop species [68,69,70,71,72,73,74]. Likewise, members of the expansin gene family play a crucial role in helping plants tolerate salt stress. These genes are involved in various physiological processes that enable plants to adapt to high salinity environments, contributing to their overall resilience and survival [75,76].

The DEGs and proteins DEPs were subjected to functional enrichment analyses using GO, COG, and KEGG pathways. Additionally, detailed expression profiling was performed. This study establishes a robust framework for advancing our understanding of the molecular mechanisms underlying cotton’s response to salt stress. Subsequent research can be tailored to create experimental conditions that thoroughly examine the entire growth cycle of plants in an authentic saline–alkali environment. This approach will comprehensively understand how various factors influence plant development in such challenging conditions.

## 4. Materials and Methods

### 4.1. Experimental Material

A selection of 430 upland cotton germplasm resources, showcasing diverse geographical origins and well-documented resequencing genome backgrounds, was utilized for this study. Among these, 385 accessions were sourced from the national mid-term cotton germplasm bank, which involved a rigorous selection process from over 3000 candidates. This selection was guided by extensive phenotypic characterization and purification efforts across multiple years, emphasizing traits such as disease resistance, heat tolerance, salinity–alkali resilience, high yield, and quality characteristics. The 45 semi-wild germlines of *G. purpurascens* are precious germplasm resources collected by the research team from the coastal islands of China (Guangdong Jinzhou Island and Techeng Island, Hainan Island and Xisha Islands) over many years.

### 4.2. Experimental Design

The ionome determination experiment was carried out in the artificial climate chamber of Zhengzhou Scientific Research Center, Cotton Research Institute, Chinese Academy of Agricultural Sciences from 2021 to 2022. The chamber had a day and night photoperiod of 14 h/10 h, a light intensity of more than 12,000 lumens, and a relative humidity of 60–70%. Each variety was cultivated in 6 pots, with 4 plants per pot. For sampling, every 8 plants constituted a biological replicate, resulting in 3 replicates for each variety. After soaking and disinfecting the cotton seeds in a 15% sodium hypochlorite solution, they were rinsed with deionized distilled water (ddH_2_O) three times. The seeds were placed in a constant-temperature climate chamber for 24 h before planting. They were cultivated using the sand and water cultivation method, maintaining a ratio of 8:1. After the cotton seedlings grew to the three-leaf one-heart stage, the seedlings with consistent growth were selected and treated with two NaCl concentrations of 0% (control) and 0.4% (salt treatment). In order to prevent the salt shock effect, the salt was added gradually three times [35]. After 72 h of treatment, the roots, stems (including petioles), and leaves (including cotyledons and true leaves) were packed into paper bags and dried in an oven at 105 °C for 20 min and then dried at 80 °C for 48 h. Their dry weight (g, DW) was accurately measured, and the ion content was determined.

The phenotypic classification experiment of salt stress was carried out in 2023 and was based on the validation experiment results of ion group experiments. The seedling planting method and salt stress concentration in this experiment were consistent with those of the ionome determination test. Still, the salt stress time was extended from three days (72 h) to 10 days (the basic death stage of salt-sensitive materials). The salt damage of plants at different time points (0 h, 48 h, 72 h, 96 h, 10 day) was graded before and after salt stress.

### 4.3. Measurement Indicators and Methods

The Thermo Scientific XSeries 2 Inductively Coupled Plasma Mass Spectrometer (Thermo Fisher Scientific, Waltham, MA, USA) was employed to assess the salt-tolerant phenotype of an upland cotton population by quantifying the concentrations of potassium (K), calcium (Ca), sodium (Na), and magnesium (Mg) ions in roots, stems, and leaves following a 72 h exposure to salt stress.

#### 4.3.1. Preparation of Sample Solution

Before each batch of samples was prepared, they were dried and accurately weighed, and the dried samples were crushed to below 40 mesh. First, take the roots and leaves with a known exact weight and place them in a beaker. Set the beaker on a plate heater and gradually heat it to completely dry and carbonize the material. Afterwards, add 1–2 mL of concentrated sulfuric acid dropwise, ensuring even soaking, and place the beaker back on the plate heater to heat slowly until all sulfuric acid vapors are removed. Next, transfer the beaker to a muffle furnace set at 550 °C for complete ashing. After the beaker has cooled to room temperature, add 2 mL of nitric acid to dissolve the contents thoroughly. Transfer the solution entirely into a 50 mL centrifuge tube. Then, rinse the beaker with 48 mL of ultrapure water to ensure all material was recovered. Finally, dilute the sample solution, and shake it well. If the absorption values of K, Na, Ca, and Mg are outside the linear range, the batch must be diluted. To do this, accurately measure 1 mL of the sample solution, and place it in a 100 mL centrifuge tube. Then, add 99 mL of a 1.5% nitric acid solution to dilute the sample. Shake the tube to ensure the sample was well mixed during the two-step dilution process.

#### 4.3.2. Measurement Method

(1) Determination Method of Sample Solution

The sample injection tube was immersed in the test solution for analysis. After the analysis, the tube was rinsed with a 1.5% nitric acid solution before proceeding with the following sample. When the high-concentration solution was lower than the low-concentration solution during the assay, rinse the sample injection tube with a 1.5% nitric acid solution for 5 min.

(2) Determination of Two-Step Dilution Sample Solution

The sample injection tube was placed in the two-step dilution sample solution for determination. After the determination was completed, the sample injection tube was rinsed with 1.5% nitric acid solution, and the two-step dilution sample solution was continued. When transitioning from a high-concentration solution to a low-concentration solution during the assay, rinse the sample injection tube with 1.5% nitric acid solution for 5 min.

#### 4.3.3. Standard Curve

The reagent blank solution and six standard solutions, each with varying concentrations, were injected separately. The readings for the elements potassium, sodium, calcium, and magnesium were recorded, resulting in a series of response values. These values were then linearly regressed against the injection concentrations (in ppm) to create a standard curve. The standard curve method was used to calculate the elemental content of potassium, sodium, calcium, and magnesium. The relative content of each ion was measured in mmol/(g, DW).

Absolute ion content=Relative content × Dry weight (DW) in mmol

The calculation formula for the determination of the sample solution:

$$Sample\;content\left(\frac{g}{kg}\right)=\frac{Sample\;concentration\;\left(ppm\right)\;\times \;50}{Weighing\;volume/1000}$$

$$Sample\;diluted\;in\;the\;2nd\;step\;\left(\frac{g}{kg}\;\right)=\;\frac{Sample\;concentration\;\left(ppm\right)\;\times \;50\;\times \;100}{Weighing\;volume/1000}$$

The relative content of each ion was measured in mmol/(g, DW).

Absolute ion content = Relative content × Dry weight (DW) in mmol

To remove the influence of different material genotypes, the salt tolerance coefficient (ST) was introduced to measure the salt tolerance of cotton seedlings. The ST measures the ratio of the phenotypic value under salt stress to that of the control.

### 4.4. Phenotypic Data Calculation

The calculation indicators for ion group-related traits primarily include the relative content of four ions: Ca, K, Mg, and Na. This relative content is expressed in mol/(kg, DW) units and converted to mmol/(g, DW). Additionally, comparisons were made between K vs. Na, Ca vs. Na, and Mg vs. Na in various tissues or parts of cotton. Other important metrics include the salt tolerance coefficient (ST), which is dimensionless, the ion transport selectivity coefficient (ISC), and the relative selectivity transport coefficient (RISC), which is dimensionless. These coefficients were evaluated for transport from underground to aboveground parts, stem to leaf, and root to stem. The calculation formula is as follows:

Relative content of the four ions:

(1) $$RC=\frac{Na/K/Ca/Mg\;(mg\;or\;mmol)}{Sample\;dry\;weight\;DW(g)}$$

Salt tolerance coefficient of each index:

(2) $$ST=\frac{Relative\;content\;of\;salt\;treatment\;group}{Relative\;content\;of\;the\;control\;group}\;\times \;100$$

Selectivity coefficient of ion transport:

(3) ISC−Sh_R(X−Na)=Above ground parts(X−Na)/Root(X−Na)

(4) ISC−L_S(X−Na)=Leaf(X−Na)/Stem(X−Na)

(5) ISC−S_R(X−Na)=Stem(X−Na)/Root(X−Na)

Relative selectivity transport coefficient of ions:

(6) $$\begin{array}{c} RISC-Sh\_R(X-Na)\\ =Salt\;treatment\;ISC-Sh\_R(X-Na)/Control\;ISC-Sh\_R(X-Na) \end{array}$$

(7) $$\begin{array}{c} RISC-L\_S(X-Na)\\ =Salt\;treatment\;ISC1-L\_S(X-Na)/Control\;ISC-L\_S(X-Na) \end{array}$$

(8) $$\begin{array}{c} RISC-S\_R(X-Na)\\ =Salt\;treatment\;ISC-S\_R(X-Na)/Control\;ISC-S\_R(X-Na) \end{array}$$

where X represents any of the three ions K, Ca, and Mg, and ISC is calculated using the relative content of the ions. The relative selectivity transport coefficient of ions (RISC) is the salt tolerance coefficient of the ion-selective transport coefficient (ISC).

Membership function values of the indicator:

(9) $$U(X_{r})=\left(X_{r}-X_{min}\right)/{(X}_{max}-X_{min})\;r\;=\;1,\;2,\;3,\;\ldots ,\;n$$

(10) $$U(X_{r})=\left(X_{max}-X_{r}\right)/{(X}_{max}-X_{min})\;r\;=\;1,\;2,\;3,\;\ldots ,\;n$$

X_r_ represents the r-rd composite indicator; X_min_ represents the minimum value of the r-th composite indicator; and X_max_ represents the maximum value of the r-th composite metric. When the salt tolerance index was positively correlated with the salt tolerance ability of the germplasm, Equation (9) was used. When the salt tolerance index was negatively correlated with the salt tolerance capacity of the germplasm, Equation (10) was used.

Weights of the indicator:

(11) $$W_{r}=\frac{Pr}{\sum_{r=1}^{n}Pr}\;r\;=\;1,\;2,\;3,\;\ldots ,\;n$$

W_r_ indicates the importance of the r-rd composite indicator among all composite indicators, i.e., the weight; Pr represents the contribution rate of the r-rd comprehensive index of each cotton variety obtained by principal component analysis.

Comprehensive salt tolerance index (CSTI):

(12) $$CSTI=\sum_{r=1}^{n}\left[U(X_{r})\times H_{r}\times W_{r}\right]\;r\;=\;1,\;2,\;3,\;\ldots ,\;n$$

The CSTI value (comprehensive salt tolerance index) is the salt tolerance evaluation value of different cotton varieties under salt stress conditions. H_r_ represents the load of the r-rd composite index of each cotton variety obtained by principal component analysis.

### 4.5. TMT Quantitative Proteomics

Two upland cotton materials were selected from a population of 430 upland cotton lines: the salt-tolerant variety Ejing92 (K069; E9) and the salt-tolerant control variety Z9807 (K372; Z9). The Z9807 material was developed through pedigree breeding techniques, with extensive characterization and analysis conducted by researcher Ye Wuwei at the Cotton Research Institute of the Chinese Academy of Agricultural Sciences. This work has been documented over several years, mainly focusing on methodologies for identifying salt tolerance within cotton germplasms. This genetic material is conserved within the gene bank at the Cotton Research Institute and is presently being disseminated by the project team focused on the characterization of stress resistance in cotton varieties. This study identified and selected the salt-tolerant variety Ejing92 based on a comprehensive assessment of its salt stress phenotypic response and ionomic profile.

#### 4.5.1. Growth Conditions and Treatment

Cotton seedlings were grown using the sand culture method in an artificial climate chamber until they reached the three-leaf and one-heart stage. A high-concentration NaCl solution of 0.4% (4.0 g NaCl/1 kg sand) was used for salt stress treatment. The solution without NaCl was the control (0%, CK). The true leaves of both materials were collected after 12 h of salt stress and from the control group. All samples had three replicates, with 3–5 seedlings per replicate. The samples were stored in liquid nitrogen at −80 °C.

#### 4.5.2. Protein Extraction and Digestion for Proteomic Sequencing Analysis

The sample was resuspended in a lysis buffer comprising 7 M of urea (Bio-Rad, Hercules, CA, USA), 2 M of thiourea (Sigma-Aldrich, St. Louis, MO, USA), and 0.1% CHAPS (Bio-Rad). Subsequently, the tissue was mechanically disrupted using three titanium dioxide abrasive beads at 70 Hz for 120 s. This was followed by centrifugation at 5000× *g* for 5 min at 4 °C to clarify the lysate. The final supernatant was collected and stored at −80 °C until used. The Bradford protein assay measured the total protein concentration. In total, 200 μg of total protein from each sample was incubated with 5 μL of 200 mM of reducing reagent at 55 °C for 1 h. Then, 5 μL of 375 mM of Iodoacetamide was added and incubated for 10 min at room temperature in the dark. Next, 200 μL of a 100 mM dissolution buffer (AB Sciex, Foster City, CA, USA) was added and centrifuged at 12,000× *g* for 20 min. The sample was digested in trypsin for 14 h at room temperature and then lyophilized and redissolved with a 100 mM dissolution buffer for labeling.

#### 4.5.3. TMT Labeling

After the incubation of TMT reagents, 41 μL of absolute ethyl alcohol was added to the TMT reagent (0.8 mg/tube), and the mixture was mixed well. Next, 41 μL of the TMT reagent was added to 100 μg of the digested sample. The combination was then oscillated, centrifuged, and incubated for 1 h at room temperature. Following this, 8 μL of a 5% quenching reagent was added to terminate the reaction, and the mixture was incubated for an additional 15 min. Finally, the samples were stored after lyophilization.

#### 4.5.4. Peptide Identification by Nano UPLC-MS/MS

The peptide fractions obtained were reconstituted in 20 μL of buffer A (comprising 0.1% formic acid and 2% acetonitrile), followed by centrifugation at 12,000 rpm for 10 min. Subsequently, 10 μL of the resulting supernatant was injected into a nano UPLC-MS/MS system, which included a Nanoflow HPLC system (EASY-nLC 1000, Thermo Scientific, Waltham, MA, USA) coupled with an Orbitrap Fusion Lumos mass spectrometer (Thermo Scientific). The sample was loaded onto Acclaim PepMap100 C18 column and then separated by an EASY-Spray C18 column. The mass spectrometer was configured for positive ion detection, utilizing a source voltage of 2.1 kV. Full MS scans were conducted using the Orbitrap over a mass-to-charge (*m*/*z*) range of 300 to 1500, achieving a resolution of 120,000. In the MS/MS analysis, the 20 most abundant ions exhibiting multiple charge states were chosen for higher energy collisional dissociation (HCD) fragmentation, utilizing data from a single full MS scan. The database used in this experiment was the Gh-TM-1_CRI (https://www.cottongen.org/species/Gossypium_hirsutum/CRI-AD1_v1; accessed on 20 June 2024) database. The resulting MS/MS data were processed using Proteome Discoverer 1.4.

#### 4.5.5. Protein Identification

Protein identification parameters were established: precursor ion mass tolerance was set to ±15 ppm, while fragment ion mass tolerance was maintained at ±0.5 Da. A maximum of two missed cleavages were allowed. Static modifications included carboxyamidomethylation (57.021 Da) of cysteine residues. For dynamic modifications, the oxidation of methionine residues was accounted for (+15.995 Da). After analyzing the primary data, only those entries with a *p*-value ≤ 0.05 and a differential ratio ≥ 2 were subjected to further analysis.

### 4.6. Data Processing

All data were processed and statistically analyzed using Microsoft Excel and IBM SPSS Statistics 26 software. For the principal component analysis, the key traits related to salt tolerance in the upland cotton ionome phenotype were standardized. A correlation matrix was created and evaluated, which allowed us to obtain characteristic values and relative contribution rates. Additionally, the factor scores for each principal component across the different varieties were calculated [77,78]. The CSTI was employed in cluster analysis as the distance metric for germplasms within the test population. The shortest distance method was utilized as the clustering technique to create the cluster dendrogram of the germplasm resources [77,79].

## 5. Conclusions

This study assessed the salt tolerance of 430 cotton germplasm resources at a 0.4% NaCl concentration. We transformed 43 individual indexes into 7 new comprehensive indexes using correlation analysis, principal component analysis, a comprehensive membership function, and cluster analysis. Based on this analysis, we determined the overall salt tolerance values for the 430 cotton germplasms. Correlation analysis showed that the relative content of K/Ca/Mg/Na in stems and leaves was closely related to salt tolerance. Based on the CSTI value, the germplasm was categorized into five groups. The phenotypic classification of salt damage after salt stress at the seedling stage for all varieties was reassessed. Ultimately, 30 salt-tolerant, 114 weakly salt-tolerant materials, 39 salt-sensitive materials, and 4 highly sensitive materials were identified. Using TMT-based quantitative analysis, 3107 and 28,642 peptides were identified, with 203,869 secondary mass spectra, of which 50,039 were matched to peptides. Moreover, salt tolerance-related proteins were also identified as CAZy family members. This methodology will facilitate a thorough investigation into the interplay of diverse environmental and physiological factors that impact plant development under adverse conditions.

## Figures and Tables

**Table 1:** Grading criteria for salt tolerance of cotton seedlings after salt stress (salt damage phenotype).

Grade	Salt-Tolerant Grade	Phenotypic Characteristics	Number of Materials	Frequency (%)
A1	Salt-resistant	No wilting occurred after salt stress treatment. On the 10th day after salt stress, the growth was still normal, and there were basically no symptoms of salt damage.	2	0.47
A2	Salt-tolerant	There was no obvious wilting of leaves 6 h after salt stress. A few leaves appeared wilted or drooping at 96 h after salt stress. On the 10th day after salt stress, a few leaves showed yellowing.	93	21.63
A3	Weakly salt-tolerant	There was no obvious wilting of leaves 6 h after salt stress. After salt stress, about half of the leaf area appeared wilted or drooped at 96 h. On the 10th day after salt stress, about half of the leaves turned yellow.	199	46.28
A4	Salt sensitivity	Leaf wilting was obvious at 96 h after salt stress. On the 10th day after salt stress, most of the leaf area dried up and lost water, and the growth was seriously hindered.	92	21.39
A5	Highly sensitive	Leaf wilting was obvious at 96 h after salt stress. On the 10th day after salt stress, the plants were severely damaged, and the leaves and stems were basically completely wilted.	44	10.23

**Table 2:** Classification and frequency of salt tolerance grades in cotton seedlings under salt stress.

Grade	Salt Resistance	CSTI	Number of Materials	Frequency (%)	Proportion of Gp Cotton (%)
L1	Salt-resistant (SR)	5.633	1	0.23	0
L2	Salt-tolerant (ST)	3.652–5.108	71	16.51	0
L3	Weakly salt-tolerant (WST)	2.503–3.629	220 (28)	51.16	12.73
L4	Salt sensitivity (SS)	1.563–2.478	119 (15)	27.67	12.61
L5	Highly sensitive (HSS)	0.411–1.495	19 (2)	4.42	10.53

**Table 3:** Phenotypic evaluation of ion group and cross-grading statistics of salt damage phenotype.

Phenotypic Grading	A1	A2	A3	A4	A5	Total
L1			1			1
L2		30	37	4		71
L3	1	47	114	38	20	220
L4	1	16	43	39	20	119
L5			4	11	4	19
Total	2	93	199	92	44	430

## Data Availability

The datasets presented in this study can be found in online repositories. The mass spectrometry proteomics data were deposited to the ProteomeXchange Consortium via the PRIDE partner repository with the dataset identifier PXD058796.

## Supplementary Materials

The supporting information can be downloaded at https://www.mdpi.com/article/10.3390/ijms26051982/s1.

## References

[1] Munns R., Tester M. (2008). Mechanisms of salinity tolerance. Annu. Rev. Plant Biol..

[2] Farooq T., Zain-ul-Hudda Q.F., Fatima R., Muneer R., Ali A., Shehzad S., Imran A., Sohail S., Iftikhar A., Mazhar M. (2024). Overexpression of gene ΔGH_A07G1537 associated with fiber quality in upland cotton (*Gossypium hirsutum* L.) Through pollen tube transformation method. Agrobiol. Rec..

[3] Gupta B., Huang B. (2014). Mechanism of Salinity Tolerance in Plants: Physiological, Biochemical, and Molecular Characterization. Int. J. Genom..

[4] Yichie Y., Brien C., Berger B., Roberts T.H., Atwell B.J. (2018). Salinity tolerance in Australian wild *Oryza* species varies widely and matches that observed in *O-sativa*. Rice.

[5] Qin X., Duan Z. (2019). Signal Regulation Mechanism of Plant Salt Stress. Genom. Appl. Biol..

[6] Gerona M.E.B., Deocampo M.P., Egdane J.A., Ismail A.M., Dionisio-Sese M.L. (2019). Physiological Responses of Contrasting Rice Genotypes to Salt Stress at Reproductive Stage. Rice Sci..

[7] Chinnusamy V., Zhu J., Zhu J.-K. (2006). Salt stress signaling and mechanisms of plant salt tolerance. Genet. Eng..

[8] Hosseini M.K., Powell A.A., Bingham I.J. (2002). Comparison of the seed germination and early seedling growth of soybean in saline conditions. Seed Sci. Res..

[9] Etukuri S.P. (2021). A Comparative Gene Expression and Regulation Analyses Among Four Gossypium Species. Ph.D. Thesis.

[10] Zafar M., Ijaz A., Anwar Z., Shakeel A., Qadir F., Kamal H., Razzaq A., Jiang X. (2024). Ancient to modern origins: The evolutionary journey of *Gossypium* genus and its implications for cotton breeding. Agrobiol. Rec..

[11] Xu Y., Wei Y., Li Z., Cai X., Wang Y., Wang X., Zhang Z., Wang K., Liu F., Zhou Z. (2018). Integrated Evaluation and the Physiological and Biochemical Responses of Semi-Wild Cotton under Complex Salt-Alkali Stress. Cotton Sci..

[12] Nazir M.F., He S., Ahmed H., Sarfraz Z., Jia Y., Li H., Sun G., Iqbal M.S., Pan Z., Du X. (2021). Genomic insight into the divergence and adaptive potential of a forgotten landrace *G. hirsutum* L. *purpurascens*. J. Genet. Genom..

[13] Cai C., Zhu G., Zhang T., Guo W. (2017). High-density 80 K SNP array is a powerful tool for genotyping *G. hirsutum* accessions and genome analysis. BMC Genom..

[14] Ding G., Liu J., Shi L., Xu F. (2010). Plant inomics: A new field in plant nutrition. J. Plant Nutr. Fertil..

[15] Guo X. (1995). Current status and development of special cotton breeding in China. Cotton Sci..

[16] Xu Z., Lu X., Wei Y., Meng C., Zhang M., Zhang Y., Wang M., Wang J., Zhang C., Li Y. (2023). Identification and evaluation of salt tolerance of space-induced wild soybean SP1 population at the seedling stage. Acta Prataculturae Sin..

[17] Bai D., Xue Y., Huang L., Huai D., Tian Y., Wang P., Zhang X., Zhang H., Li N., Jiang H. (2022). Identification of cold tolerance and screening of evaluation indices for different peanut varieties at the germination stage. Acta Agron. Sin..

[18] Gu X., Wu F., Liu H., Nie J., Gao H., Ma Q. (2020). Identification and evaluation of salt tolerance of 30 rice materials. J. Trop. Biol..

[19] Sun X., Jiang Q., Hu Z., Li H., Pang B., Zhang F., Zhang S., Zhang H. (2023). Identification and evaluation of salt tolerance of wheat germplasm resources at the seedling stage. Acta Agron. Sin..

[20] Hu L., Wang S., Wang L., Cheng X., Chen H. (2022). Identification of salt tolerance and screening of salt-tolerant germplasm of mung bean at the seedling stage. Acta Agron. Sin..

[21] Li P., Yan J., Zhang H., Zhang Y., Tao S., Zhang Q., Aldiyar X.A., Huang Z. (2021). Screening and evaluation of salt tolerance of 146 *Brassica napus* germplasms at the germination stage. Acta Agric. Boreali-Occident. Sin..

[22] Jamshidi Goharrizi K., Baghizadeh A., Kalantar M., Fatehi F. (2020). Assessment of changes in some biochemical traits and proteomic profile of UCB-1 pistachio rootstock leaf under salinity stress. J. Plant Growth Regul..

[23] Nazari M., Moosavi S.S., Maleki M., Jamshidi Goharrizi K. (2020). Chloroplastic acyl carrier protein synthase I and chloroplastic 20 kDa chaperonin proteins are involved in wheat (*Triticum aestivum*) in response to moisture stress. J. Plant Interact..

[24] Jamshidi Goharrizi K., Fatehi F., Nazari M., Salehi F., Maleki M. (2020). Assessment of changes in the content of sulforaphane and expression levels of *CYP79F1* and myrosinase genes and proteomic profile of *Lepidium draba* plant under water-deficit stress induced by polyethylene glycol. Acta Physiol. Plant..

[25] Long R., Gao Y., Sun H., Zhang T., Li X., Li M., Sun Y., Kang J., Wang Z., Ding W. (2018). Quantitative proteomic analysis using iTRAQ to identify salt-responsive proteins during the germination stage of two *Medicago* species. Sci. Rep..

[26] Wang D., Mu Y., Hu X., Ma B., Wang Z., Zhu L., Xu J., Huang C., Pan Y. (2021). Comparative proteomic analysis reveals that the Heterosis of two maize hybrids is related to enhancement of stress response and photosynthesis respectively. BMC Plant Biol..

[27] Aranjuelo I., Molero G., Erice G., Avice J.C., Nogues S. (2011). Plant physiology and proteomics reveals the leaf response to drought in alfalfa (*Medicago sativa* L.). J. Exp. Bot..

[28] Rahman M.A., Yong-Goo K., Iftekhar A., Liu G.-s., Hyoshin L., Joo L.J., Byung-Hyun L. (2016). Proteome analysis of alfalfa roots in response to water deficit stress. J. Integr. Agric..

[29] Xiong J., Sun Y., Yang Q., Tian H., Zhang H., Liu Y., Chen M. (2017). Proteomic analysis of early salt stress responsive proteins in alfalfa roots and shoots. Proteome Sci..

[30] Li W., Wei Z., Qiao Z., Wu Z., Cheng L., Wang Y. (2013). Proteomics analysis of alfalfa response to heat stress. PLoS ONE.

[31] Zhang C., Shi S. (2018). Physiological and proteomic responses of contrasting alfalfa (*Medicago sativa* L.) varieties to PEG-induced osmotic stress. Front. Plant Sci..

[32] Zhang D., Yang Z., Song X., Zhang F., Liu Y. (2022). TMT-based proteomic analysis of liquorice root in response to drought stress. BMC Genom..

[33] Xiao S., Liu L., Zhang Y., Sun H., Zhang K., Bai Z., Dong H., Liu Y., Li C. (2020). Tandem mass tag-based (TMT) quantitative proteomics analysis reveals the response of fine roots to drought stress in cotton (*Gossypium hirsutum* L.). BMC Plant Biol..

[34] Pagel O., Kollipara L., Sickmann A. (2021). Tandem mass tags for comparative and discovery proteomics. Quantitative Methods in Proteomics.

[35] Peng Z., He S., Sun J., Xu F., Jia Y., Pan Z., Wang L., Du X. (2014). Efficient identification method of salt tolerance of upland cotton at the seedling stage. Acta Agron. Sin..

[36] Baxter I.R., Ziegler G., Lahner B., Mickelbart M.V., Foley R., Danku J., Armstrong P., Salt D.E., Hoekenga O.A. (2014). Single-Kernel Ionomic Profiles Are Highly Heritable Indicators of Genetic and Environmental Influences on Elemental Accumulation in Maize Grain (*Zea mays*). PLoS ONE.

[37] Li C., Dong Z., Guan Y., Liu J., Li H., Mei Y. (2024). Genetic contribution and decision coefficient analysis of agronomic characters and lint yield traits of upland cotton in southern Xinjiang. Acta Agron. Sin..

[38] Teo H.M., Aziz A., Wahizatul A.A., Bhubalan K., Nordahliawate S.M.S., Syazlie M.C.I., Ng L.C. (2022). Setting a Plausible Route for Saline Soil-Based Crop Cultivations by Application of Beneficial Halophyte-Associated Bacteria: A Review. Microorganisms.

[39] Han X., Wu Z., Liu F., Wang Y., Wei X., Tian P., Ling F. (2023). Transcriptomic Analysis and Salt-Tolerance Gene Mining during Rice Germination. Genes.

[40] Peng Z., Rehman A., Li X., Jiang X., Tian C., Wang X., Li H., Wang Z., He S., Du X. (2023). Comprehensive Evaluation and Transcriptome Analysis Reveal the Salt Tolerance Mechanism in Semi-Wild Cotton (*Gossypium purpurascens*). Int. J. Mol. Sci..

[41] Liu G., Lu L., Liu J. (1993). Identification of salt tolerance of cotton variety resources. Crop Var. Resour..

[42] Sun X., Liu Y., Chen Q. (1998). Research progress on salt tolerance of cotton. Cotton Sci..

[43] Wang J., Wang D., Fan W., Song G., Wang S., Ye W. (2011). The characters of salt-tolerance at different growth stages in cotton. Acta Ecol. Sin..

[44] Xiong S., Wang Y., Chen Y., Gao M., Zhao Y., Wu L. (2022). Effects of Drought Stress and Rehydration on Physiological and Biochemical Properties of Four Oak Species in China. Plants.

[45] Zhang R., Hussain S., Wang Y., Liu Y., Li Q., Chen Y., Wei H., Gao P., Dai Q. (2021). Comprehensive Evaluation of Salt Tolerance in Rice (*Oryza sativa* L.) Germplasm at the Germination Stage. Agronomy.

[46] Zhou Q., Lu Q., Zhao Z., Wu C., Fu X., Zhao Y., Han Y., Lin H., Chen W., Mou L. (2024). Identification of drought tolerance of 244 spring wheat varieties at the seedling stage. Sci. Agric. Sin..

[47] Sun J.-b., Sun G.-y., Liu X.-d., Hu Y.-b., Zhao Y.-s. (2009). Effects of salt stress on mulberry seedlings growth, leaf water status, and ion distribution in various organs. Ying Yong Sheng Tai Xue Bao J. Appl. Ecol..

[48] Zhang J., Li J., Song Y., Xing S., Chi J., Ma B. (2003). Advances in Research on the Mechanism of Plant Salinity Tolerance and Breeding of Salt-tolerant Plants. World For. Res..

[49] Wu H., Su W., Shi M., Xue X., Ren H., Wang Y., Zhao A., Li D. (2022). Diversity Analysis and Comprehensive Evaluation of Jujube Fruit Traits. J. Plant Genet. Resour..

[50] Zhang X., Chen C., Zhang J., Zeng Y., Bao M., Zhang S., Shang J., Sha X., Wu J., Zhang G. (2023). Analysis and Comprehensive Evaluation of Agronomic and Yield Traits of 55Alfalfa Varieties. Acta Agrestia Sin..

[51] Ma X., Liu Y., Shang W., Gao N., Zhang T., Piao F., Wang Y., Zhao W. (2024). Comprehensive evaluation of salt tolerance of 44 watermelon germplasm resources at the seedling stage. China Cucurbits Veg..

[52] Ma S., Tian R., Hu H., Lü J., Tian L., Luo C., Zhang Y., Li P. (2020). Comprehensive evaluation and screening of salt tolerance of japonica rice germplasm resources at the seedling stage. J. Plant Genet. Resour..

[53] Wang W., Wang W., Zou J., Yu L., Wang Z., Wang F., Lu L., Niu L. (2022). Research progress on comprehensive evaluation methods of salt tolerance in wheat. Mod. Agric. Res..

[54] Haque M.S., Hasanuzzaman M., Rahman M.T., Islam N., Begum S.N., Yasmin S. (2022). Hydroponic and in vitro screening of wheat varieties for salt-tolerance. Plant Sci. Today.

[55] Dong Y., Hu G., Yu J., Thu S.W., Grover C.E., Zhu S., Wendel J.F. (2020). Salt-tolerance diversity in diploid and polyploid cotton (*Gossypium*) species. Plant J..

[56] Joshi N., Reddy S.P.P., Kumar N., Bharadwaj C., Tapan K., Patil B., Jain P.K., Nimmy M., Roorkiwal M., Verma P. (2023). Siphoning novel sources of seedling salinity tolerance from the diverse chickpea landraces. Crop Pasture Sci..

[57] Iniative A.G. (2000). Analysis of the genome sequence of the flowering plant *Arabidopsis thaliana*. Nature.

[58] Shiu S.-H., Bleecker A.B. (2003). Expansion of the receptor-like kinase/Pelle gene family and receptor-like proteins in Arabidopsis. Plant Physiol..

[59] Piovesana S., Capriotti A.L., Cavaliere C., La Barbera G., Montone C.M., Zenezini Chiozzi R., Laganà A. (2018). Recent trends and analytical challenges in plant bioactive peptide separation, identification and validation. Anal. Bioanal. Chem..

[60] Ziemann S., van der Linde K., Lahrmann U., Acar B., Kaschani F., Colby T., Kaiser M., Ding Y., Schmelz E., Huffaker A. (2018). An apoplastic peptide activates salicylic acid signalling in maize. Nat. Plants.

[61] Tavormina P., De Coninck B., Nikonorova N., De Smet I., Cammue B.P. (2015). The plant peptidome: An expanding repertoire of structural features and biological functions. Plant Cell.

[62] Zhang H., Zhu J., Gong Z., Zhu J.-K. (2022). Abiotic stress responses in plants. Nat. Rev. Genet..

[63] Shen T., Li K., Yan R., Xu F., Ni L., Jiang M. (2023). The UDP-glucuronic acid decarboxylase OsUXS3 regulates Na^+^ ion toxicity tolerance under salt stress by interacting with OsCATs in rice. Plant Physiol. Biochem..

[64] Muslu S., Kasapoğlu A.G., Güneş E., Aygören A.S., Yiğider E., İlhan E., Aydın M. (2024). Genome-Wide Analysis of *Glutathione S-Transferase* Gene Family in *P. vulgaris* Under Drought and Salinity Stress. Plant Mol. Biol. Report..

[65] Zhang C., Wu F., Yan Q., Duan Z., Wang S., Ao B., Han Y., Zhang J. (2023). Genome-Wide Analysis of the *Rab* Gene Family in *Melilotus albus* Reveals Their Role in Salt Tolerance. Int. J. Mol. Sci..

[66] Chen X., Ding Y., Yang Y., Song C., Wang B., Yang S., Guo Y., Gong Z. (2021). Protein kinases in plant responses to drought, salt, and cold stress. J. Integr. Plant Biol..

[67] Wu Z., Zhang T., Li J., Chen S., Grin I.R., Zharkov D.O., Yu B., Li H. (2023). Genome-wide analysis of WD40 protein family and functional characterization of BvWD40-82 in sugar beet. Front. Plant Sci..

[68] Guan H., Zhang Y., Li J., Zhu Z., Chang J., Bakari A., Chen S., Zheng K., Cao S. (2024). Analysis of the UDP-Glucosyltransferase (UGT) Gene Family and Its Functional Involvement in Drought and Salt Stress Tolerance in *Phoebe bournei*. Plants.

[69] Liu Q., Yang J., Wang B., Liu W., Hua Z., Jiang H. (2022). Influence of salinity on the diversity and composition of carbohydrate metabolism, nitrogen and sulfur cycling genes in lake surface sediments. Front. Microbiol..

[70] Aiana, Chauhan H., Singh K. (2024). Glycoside hydrolases reveals their differential role in response to drought and salt stress in potato (*Solanum tuberosum*). Funct. Plant Biol..

[71] Wang Z., Li N., Xu Y., Wang W., Liu Y. (2024). Functional activity of endophytic bacteria G9H01 with high salt tolerance and anti-Magnaporthe oryzae that isolated from saline-alkali-tolerant rice. Sci. Total Environ..

[72] Noh G., Kim J.-H., Cho S.W., Kim Y.-H., Jung J.-Y., Hong W.-J., Jung K.-H., Park G., Son H.-J., Jo I.H. (2022). Overexpression of the ginseng GH18 gene confers salinity tolerance in Arabidopsis. Plant Biotechnol. Rep..

[73] Zhang K., Sun Y., Li M., Long R. (2021). CrUGT87A1, a UDP-sugar glycosyltransferases (UGTs) gene from Carex rigescens, increases salt tolerance by accumulating flavonoids for antioxidation in *Arabidopsis thaliana*. Plant Physiol. Biochem..

[74] Ma Y., Song J., Sheng S., Wang D., Wang T., Wang N., Chen A., Wang L., Peng Y., Ma Y. (2024). Genome-wide characterization of *Solanum tuberosum* UGT gene family and functional analysis of *StUGT178* in salt tolerance. BMC Genom..

[75] Jadamba C., Kang K., Paek N.-C., Lee S.I., Yoo S.-C. (2020). Overexpression of rice expansin7 (Osexpa7) confers enhanced tolerance to salt stress in rice. Int. J. Mol. Sci..

[76] Dong B., Wang Q., Zhou D., Wang Y., Miao Y., Zhong S., Fang Q., Yang L., Xiao Z., Zhao H. (2024). Abiotic stress treatment reveals expansin like A gene *OfEXLA1* improving salt and drought tolerance of *Osmanthus fragrans* by responding to abscisic acid. Hortic. Plant J..

[77] Yin D., Zhang X., Wang Y., Cui D. (2011). Principal component analysis and comprehensive evaluation of main quality traits in peanut. J. Plant Genet. Resour..

[78] Xu Y., Wu D., Luo Z., Li S., Tan X., Tan Z., Ma L., Dai Y. (2011). Application of principal component analysis in walnut selection. Chin. Agric. Sci. Bull..

[79] Chen S.C., Zhu Y.L. (2004). Subpattern-based principle component analysis. Pattern Recognit..

