# Supplementary material for: Evaluation of Salt-Tolerant Germplasms and Identification of Salt Tolerance-Related Proteins in Upland Cotton at the Seedling Stage
Source: Int J Mol Sci. 2025 Feb 25;26(5):1982. doi: 10.3390/ijms26051982 (PMC11900572; doi:10.3390/ijms26051982)
Supplement: Supplementary file 1 [file ijms-26-01982-s001.zip › Suppl Figure S1-S4-new.pdf]

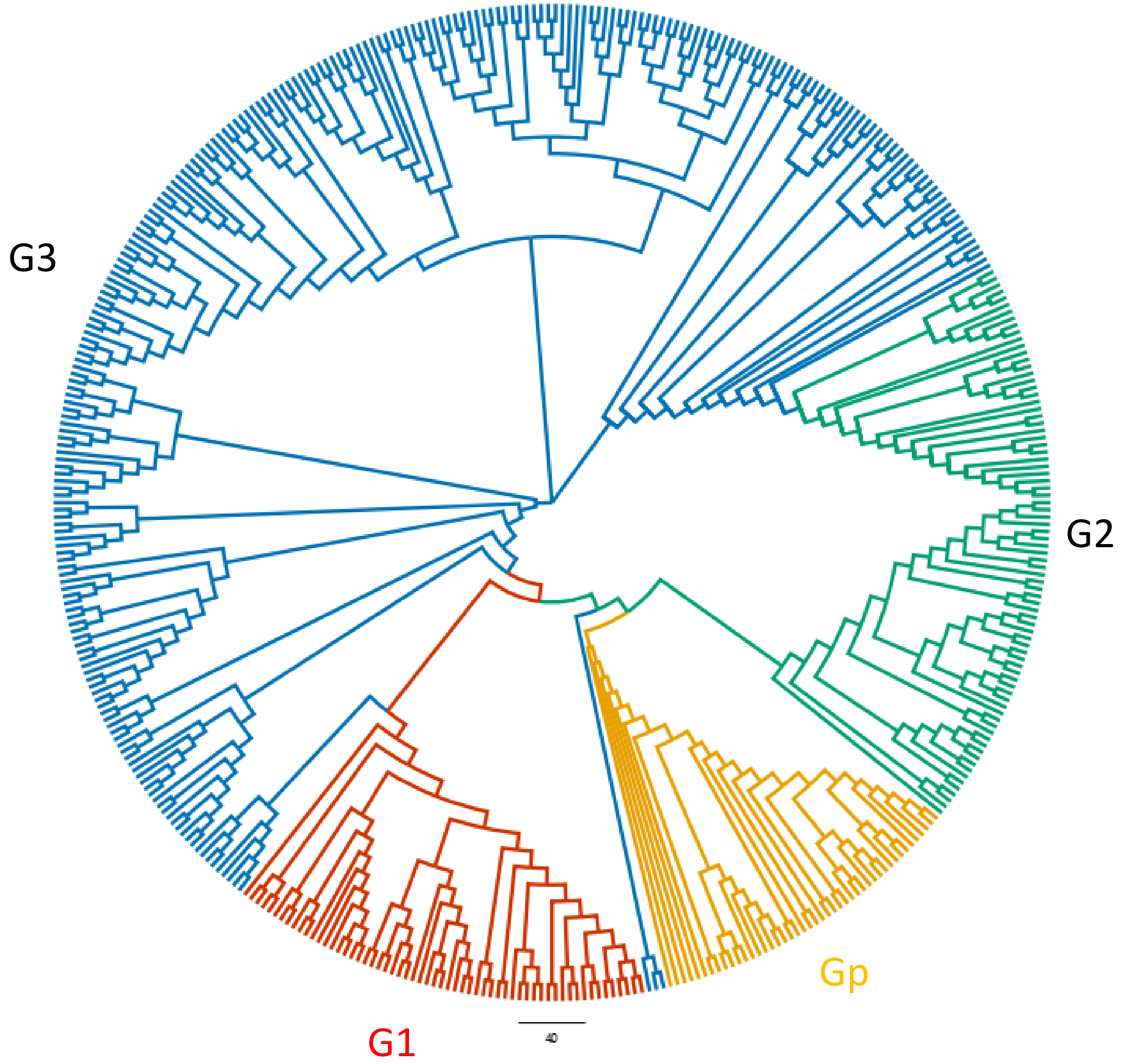

Supplementary Figure S1: Population structure and population divergence of 430 re-sequenced accessions. The accessions in Group 1 (red), Group 2 (green), Group 3 (blue) and Group p (orange) are marked by different colors.

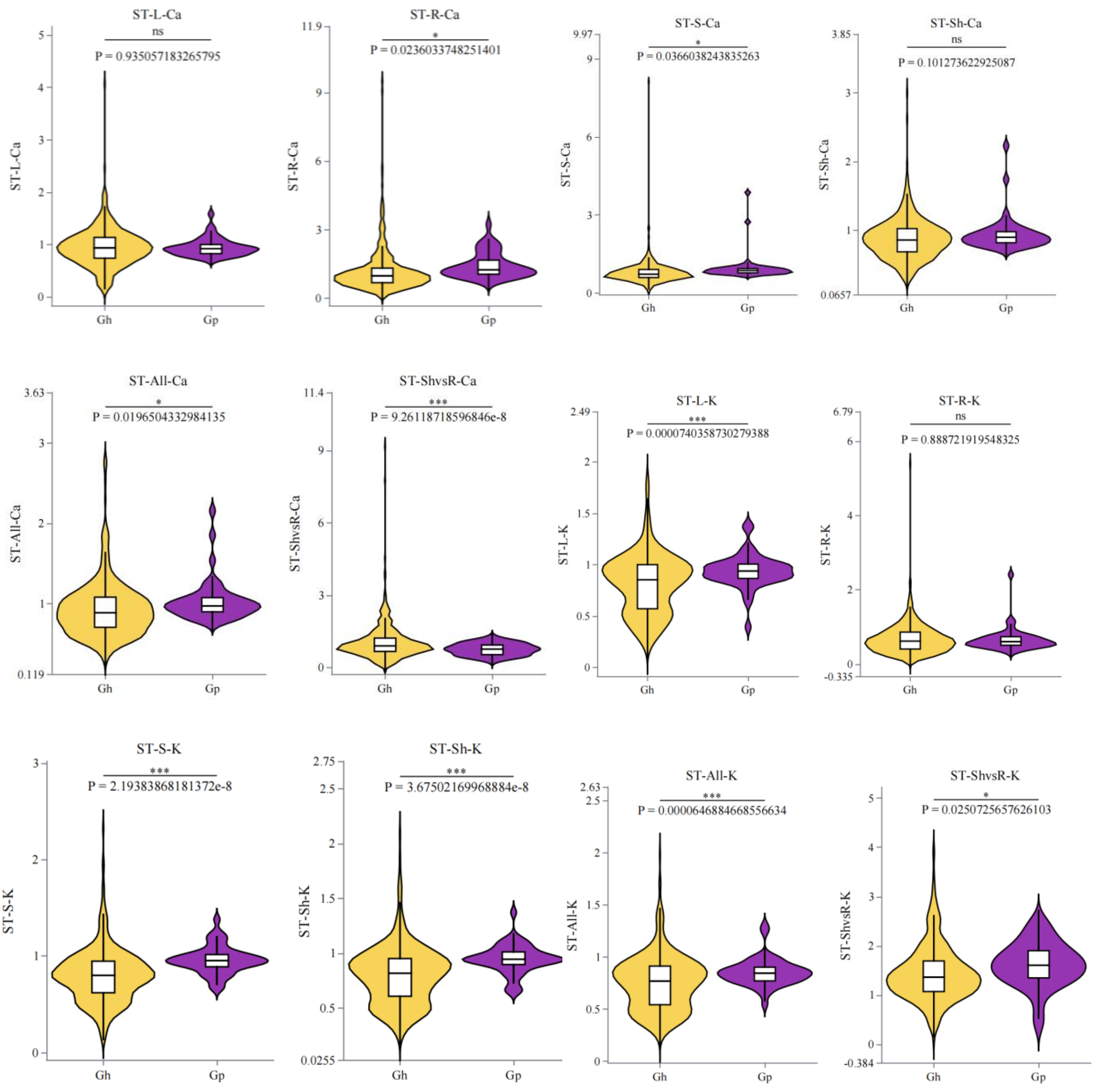

Supplementary Figure S2: Comparison of Gh vs Gp at the same salt tolerance index for Ca, K ions. Note: ns, *, ** and ***, represented *P-value>0.05*, <0.05, 0.01 ,0.001, respectively.

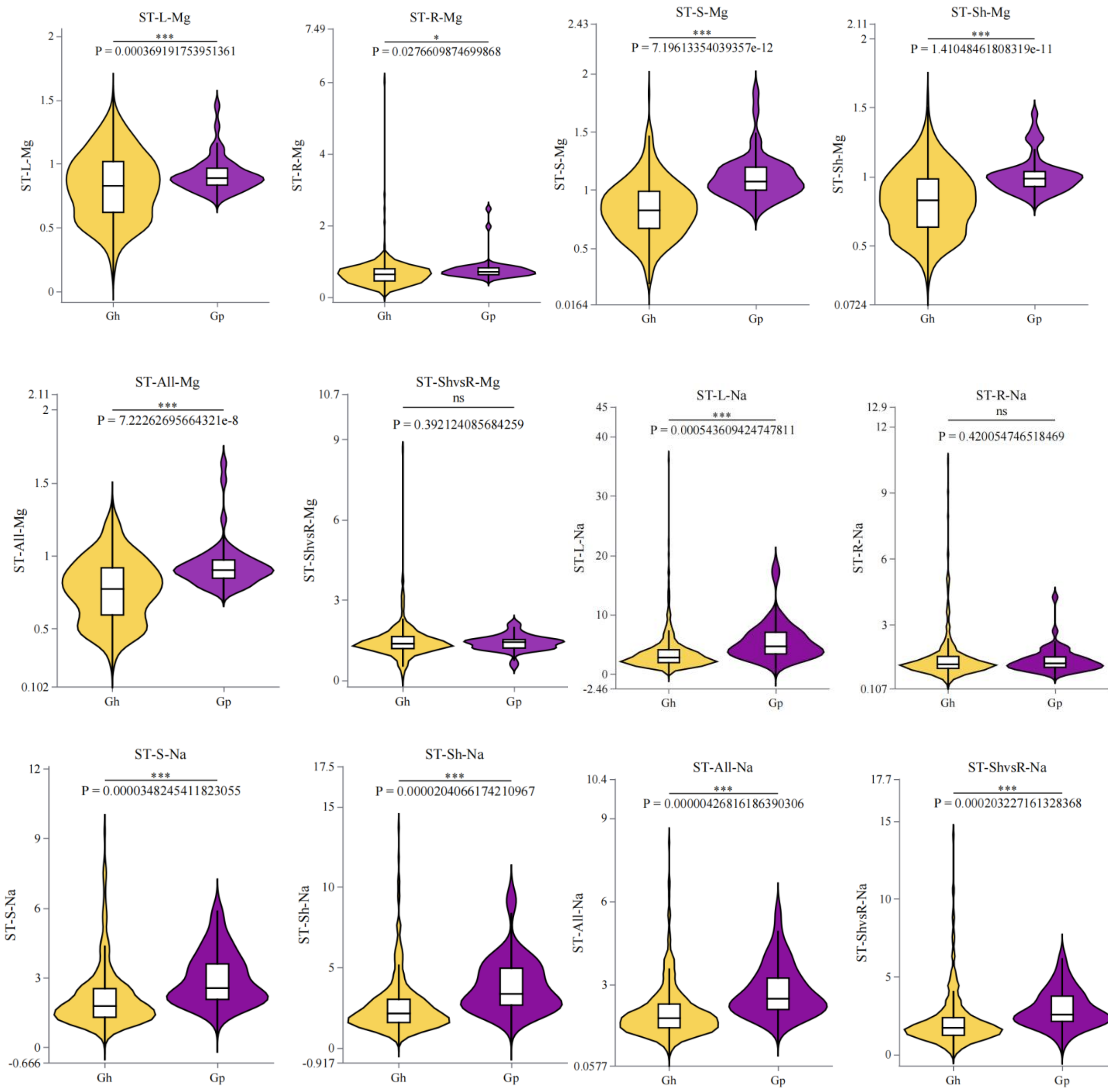

Supplementary Figure S3: Comparison of Gh vs Gp at the same salt tolerance index for Mg, Na ions. Note: ns, *, ** and ***, represented P-value>0.05, <0.05, 0.01 ,0.001, respectively.

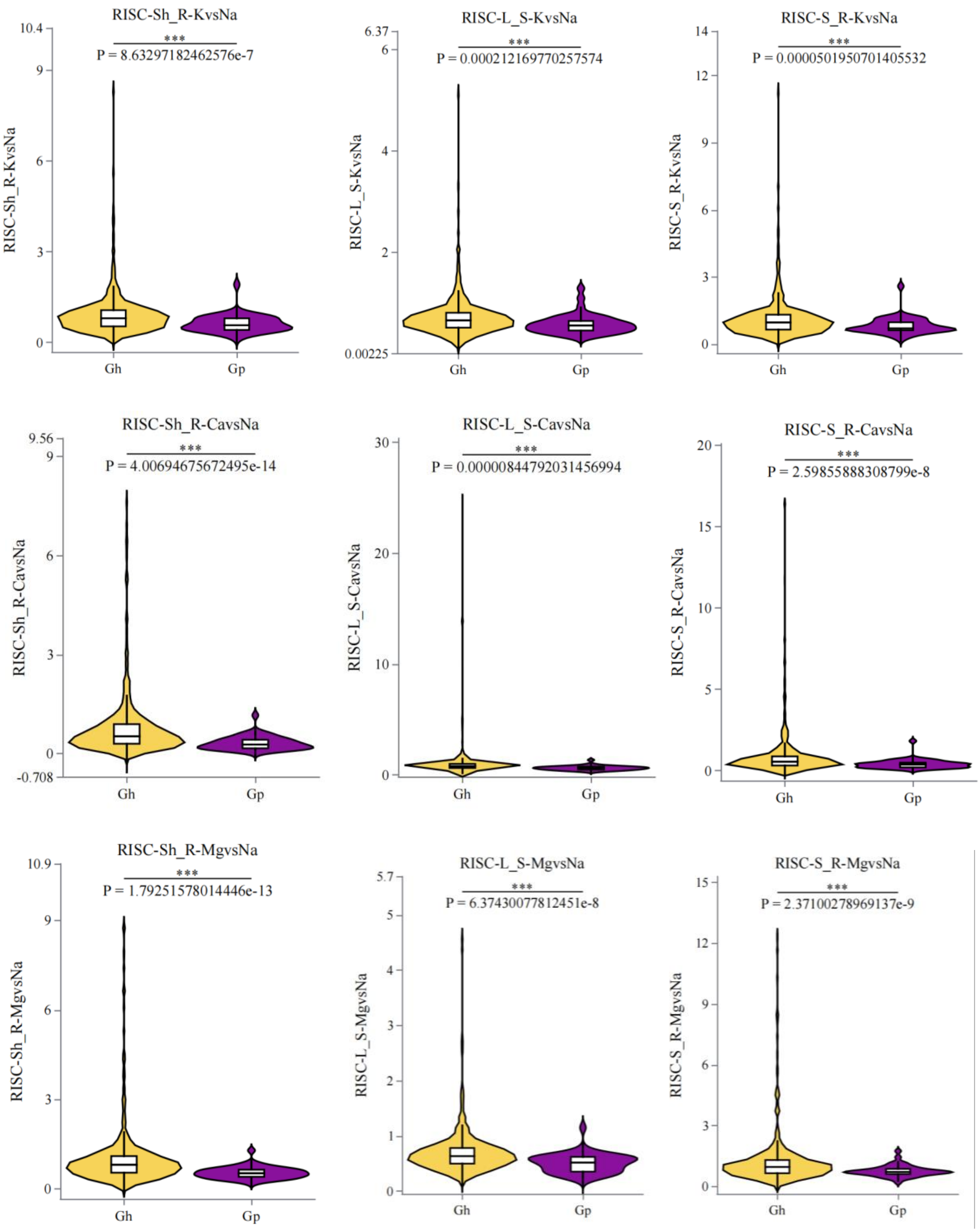

Supplementary Figure S4: Comparison of Gh vs Gp K/Na, Ca/Na, Mg/Na ion transport coefficients. Note: ns, *, ** and ***, represented P-value>0.05, <0.05, 0.01 ,0.001, respectively.
